# Advanced Electronic Packaging Technologies: A Comparative Review of Architectures, Applications, Reliability

**DOI:** 10.3390/mi17091039

**Published:** 2026-08-30

**Authors:** Yuxian Huang, Dingguan Wang, Qianyi Li, Zhiming Pan

**Affiliations:** 1 College of Electronics and Information Engineering, Shenzhen University, Shenzhen 518060, China; 2024096016@mails.szu.edu.cn (Y.H.); dgwang@szu.edu.cn (D.W.); 2 State Key Laboratory of Radio Frequency Heterogeneous Integration, Shenzhen University, Shenzhen 518060, China; 3 College of Physics and Optoelectronic Engineering, Shenzhen University, Shenzhen 518060, China; 2510232035@mails.szu.edu.cn

**Keywords:** advanced electronic packaging, system-in-package (SiP), heterogeneous integration, reliability, 2D/2.5D/3D integration

## Abstract

As transistor scaling approaches physical and economic limits, advanced packaging has become an important approach to continued system scaling. This review compares two-dimensional (2D), two- and-a-half-dimensional (2.5D), and three-dimensional (3D) integration technologies, including silicon interposers, localized silicon bridges, redistribution layer (RDL) fan-out platforms, and vertical die stacking. The comparison focuses on interconnect geometry, bandwidth, energy efficiency, thermal and mechanical constraints, manufacturing maturity, cost, and major failure mechanisms. Representative applications in power electronics, high-performance computing (HPC), artificial intelligence (AI), radio frequency (RF) systems, and micro-electromechanical systems (MEMS) are discussed together with their packaging requirements. The relationships between package structure and thermal, mechanical, and electrical reliability are also examined. Emerging technologies, including vertical power delivery, glass substrates, hybrid bonding, and AI-assisted multiphysics design, are further discussed in terms of their role in future heterogeneous integration. Finally, a near-, medium-, and long-term roadmap is presented to summarize the main scaling targets and qualification requirements for larger, denser, and higher-power integrated systems.

## 1. Introduction

As Moore’s law in integrated circuits approaches its physical limits, continued improvements in performance and energy efficiency can no longer rely solely on transistor scaling [1,2,3]. Meanwhile, applications such as high-performance computing, mobile devices, and communication systems are imposing increasingly stringent requirements on computing capability, energy efficiency, system size, and reliability. The continuous increase in chip power density further complicates thermal management and system integration [4,5]. These challenges are driving system scaling beyond the device level and toward package and system integration. In this context, advanced packaging technologies have become important enablers for sustaining the performance evolution of integrated circuits [6,7,8].

Advanced packaging enables the integration of multiple chips and functional modules within a compact package, allowing system performance and energy efficiency to be improved without relying exclusively on further transistor scaling. According to the integration dimension and interconnect structure, current approaches mainly include two-dimensional (2D), two-and-a-half-dimensional (2.5D), and three-dimensional (3D) integration. Representative implementations include silicon interposers, localized silicon bridges, redistribution layer (RDL) fan-out platforms, and vertical die stacking. Compared with conventional single-chip packaging, these technologies offer higher integration density and greater flexibility in electrical interconnection, thermal design, and system integration. In particular, System-in-Package (SiP) technology enables logic chips, memory, sensors, power devices, and other heterogeneous components to be integrated within the same package, providing more design freedom at the system level.

Higher integration density, however, also introduces a series of engineering challenges. Compact package structures and increasing power density can cause localized heat accumulation and larger temperature gradients, placing greater demands on heat spreading, cooling structures, and material selection. The use of multiple materials and stacked layers also introduces thermo-mechanical mismatch, which may lead to warpage, stress concentration, interfacial damage, and long-term reliability degradation. At high operating frequencies and data rates, parasitic effects and electromagnetic coupling associated with dense interconnects can further affect signal integrity, power integrity, and system stability. Therefore, the development of advanced packaging requires not only higher integration density but also careful consideration of electrical, thermal, mechanical, manufacturing, and reliability constraints.

Existing reviews have provided important foundations for understanding these technologies, but they generally focus on different parts of the field. Lau and co-workers documented 3D integration, fan-out packaging, TSV-less interposers, and heterogeneous integration [9,10,11,12,13,14,15], while Wang et al. connected SiP applications with reliability considerations [7]. These studies provide broad coverage of packaging technologies and their development. However, direct comparison among different integration architectures remains difficult because application requirements, manufacturing maturity, cost, reliability concerns, and reported performance metrics are often discussed separately or under different evaluation conditions.

This review therefore compares major 2D, 2.5D, and 3D integration technologies from several practical aspects, including interconnect geometry, bandwidth, energy efficiency, thermal and mechanical constraints, manufacturing maturity, cost, and dominant failure mechanisms. Representative applications in power electronics, high-performance computing, artificial intelligence, radio-frequency systems, and micro-electromechanical systems are discussed together with the requirements of different package architectures. The review also covers emerging technologies such as vertical power delivery, glass-based substrates, hybrid bonding, and AI-assisted multiphysics design, with emphasis on their roles in future heterogeneous integration. The distinct contribution of the present review is clarified below through a direct discussion of prior review scope.

Prior reviews provide complementary but differently bounded perspectives. Lau’s 2014 review emphasized 3D-IC packaging and integration [9]; the 2018 review focused on fan-out WLP for heterogeneous integration [10]; the 2019 and 2023 works broadened the discussion to heterogeneous integration and chiplets [11,15]; and the 2022 studies examined TSV-less interposers and wider advanced-packaging trends [13,14]. Wang et al. linked SiP applications to reliability [7] but offered less cross-platform quantification of manufacturing and cost. The present review, therefore, adds an architecture-application-reliability co-analysis, reports interconnect pitch, bandwidth density, energy per bit, and thermal resistance, and organizes scaling requirements into near-, medium-, and long-term horizons.

The remainder of this review is organized as follows. Section 2 introduces the main integration dimensions, interconnect structures, and commercial platforms, followed by a comparison of their major characteristics. Section 3 discusses representative applications and the suitability of different packaging architectures. Section 4 focuses on thermal, mechanical, and electrical reliability, including major failure mechanisms, evaluation methods, and mitigation approaches. Section 5 summarizes the future development of advanced packaging through near-, medium-, and long-term scaling targets for larger, denser, and higher-power heterogeneous integrated systems.

## 2. Advanced Packaging Technologies

Advanced packaging technology is one of the key technological pathways for sustaining system performance evolution in the post–Moore’s Law era, with its core objective being to achieve higher system performance, energy efficiency, and functional density through structural innovations and heterogeneous integration at the packaging level, without relying on continued transistor scaling. System-in-Package (SiP) is not a single packaging process but rather a system-integration-oriented packaging paradigm, which can be realized through different integration dimensions and interconnection schemes.

**Figure 1:**
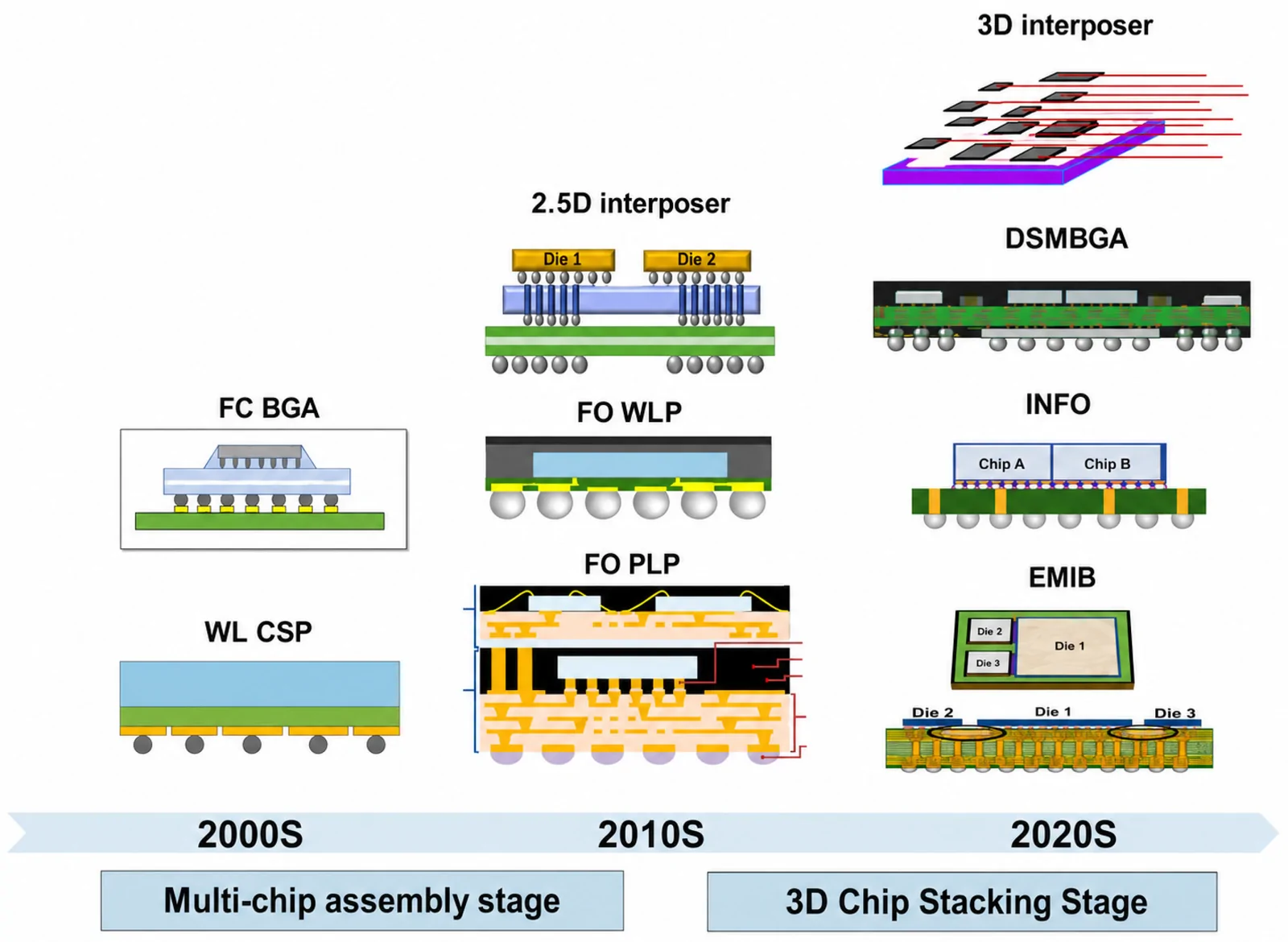
Types and development trends of advanced packaging technologies.

From the perspectives of structural configuration and integration dimension, current advanced packaging technologies can be systematically classified into three major categories: two-dimensional (2D), two-and-a-half-dimensional (2.5D), and three-dimensional (3D) packaging. As illustrated in Figure 1, different packaging technologies exhibit clear evolutionary trends in terms of integration density, interconnection schemes, and application scenarios. Based on this classification framework, this chapter provides a systematic review and analysis of representative advanced packaging technologies.

### 2.1. Two-Dimensional (2D) System-in-Package (SiP)

Two-dimensional advanced packaging technologies represent the most mature and widely adopted class of packaging solutions in the current industry, with the fundamental characteristic of integrating multiple chips or functional units within the same planar surface and achieving electrical connections through redistribution layers or conventional interconnection methods. Such technologies offer significant advantages in terms of manufacturing maturity, cost control, and yield, making them well suited for application scenarios with limited requirements for vertical integration but continued demands for reduced form factor and increased integration density.

**Figure 2:**
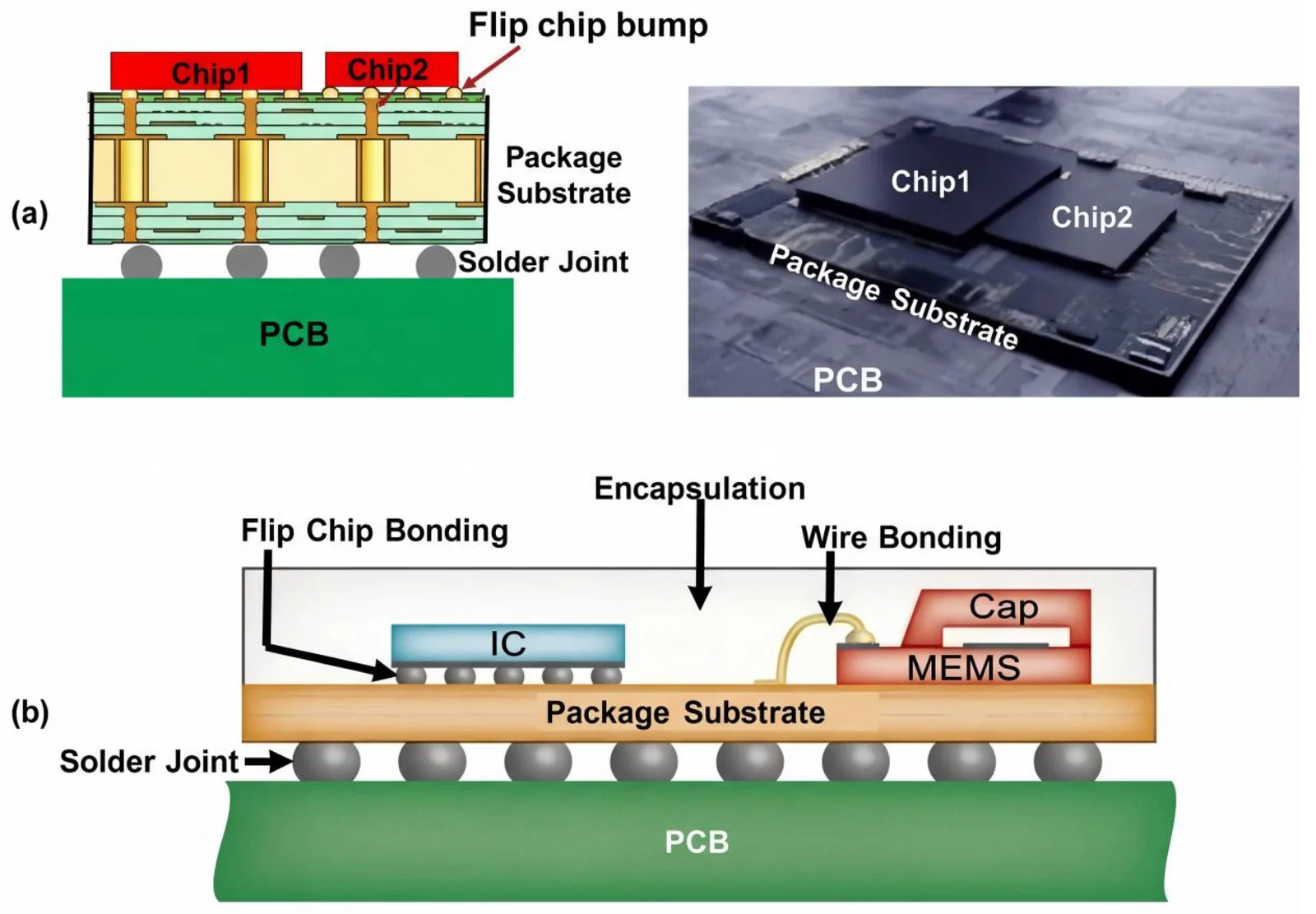
Schematic diagram of 2D packaging structures: (**a**) representative 2D package cross-section with flip-chip and wire-bonded components; (**b**) package cross-section showing chip-to-substrate interconnection and PCB mounting. Reproduced from Lau [15].

As shown in Figure 2, in a typical 2D packaging structure, multiple chips are arranged in a planar configuration on the package substrate, with interconnections between the chips and the substrate implemented via wire bonding or flip-chip techniques. This structural form is simple and intuitive, serving as a fundamental basis for understanding subsequent higher-dimensional integrated packaging technologies.

#### 2.1.1. Wafer-Level Packaging (WLP)

Wafer-Level Packaging (WLP) is an advanced packaging technology in which packaging processes are completed at the wafer level prior to dicing. Because the packaging process is performed directly on the wafer, the final package size can approach that of a bare die, offering significant advantages in miniaturization and weight reduction. Depending on the I/O expansion scheme, WLP can be mainly classified into two types: fan-in and fan-out.

Fan-in WLP has a package size that is essentially constrained by the chip itself and is suitable for applications with a relatively small number of I/Os. In contrast, fan-out WLP redistributes I/Os to the peripheral area beyond the chip footprint, effectively overcoming the limitation imposed by chip area on the number of interconnects. As the I/O count of integrated circuits continues to increase, fan-out wafer-level packaging has gradually become the mainstream solution.

**Figure 3:**
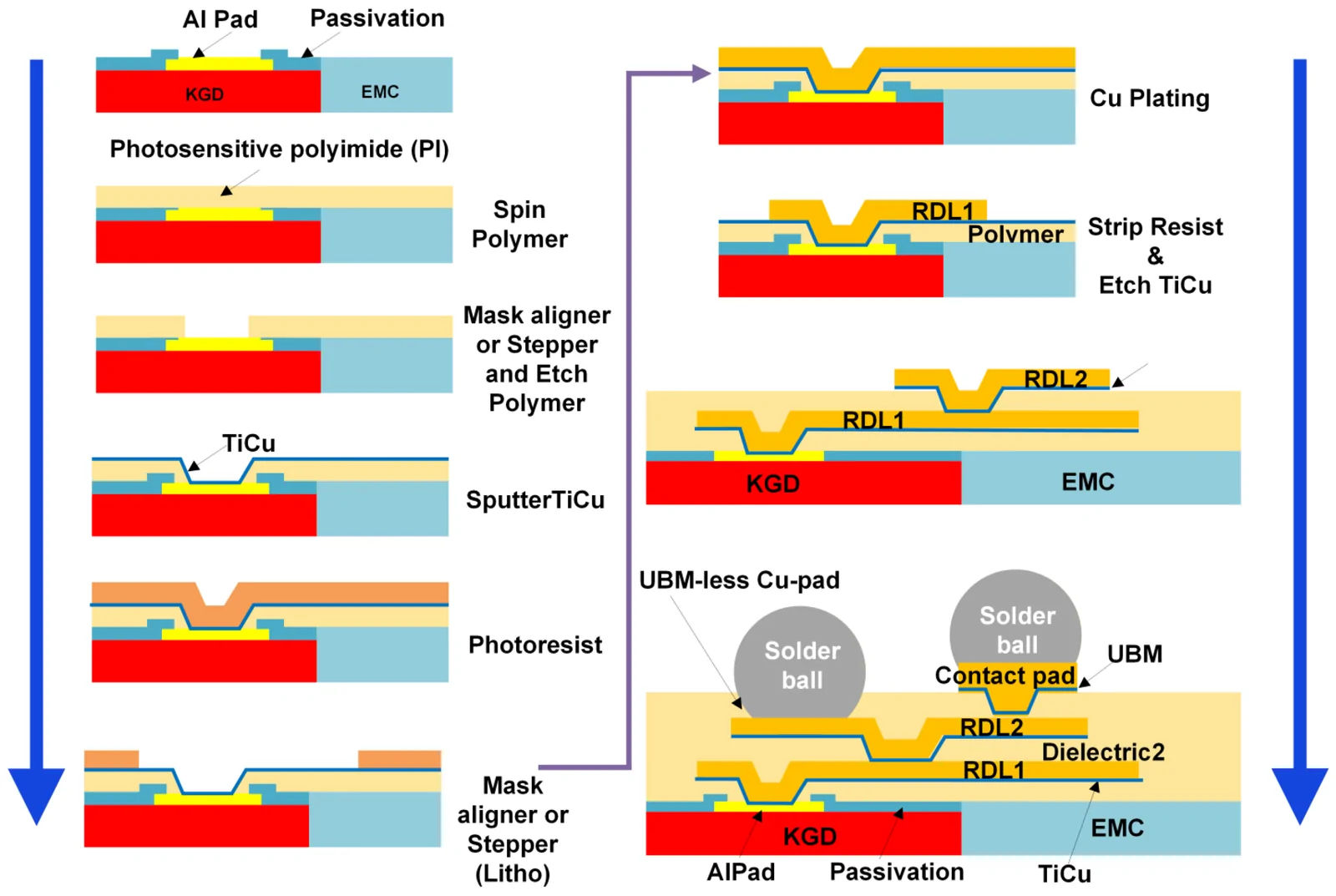
RDL process integration flow in FOWLP Adapted from Wang et al. [7].

In fan-out wafer-level packaging (FOWLP), the redistribution layer (RDL) plays a critical role in enabling high-density interconnections. As illustrated in Figure 3, the RDL process redistributes the original chip pads to a larger area through multilayer metal deposition and routing, thereby significantly increasing the I/O density of the package and effectively alleviating the issue of insufficient solder ball count caused by pin limitations in conventional packaging.

FOWLP demonstrates outstanding performance in miniaturization, thermal dissipation capability, and the balance between performance and cost and has been widely applied in mobile terminals, wearable devices, and certain medium-power applications.

#### 2.1.2. Fan-Out Panel-Level Packaging (FOPLP)

**Figure 4:**
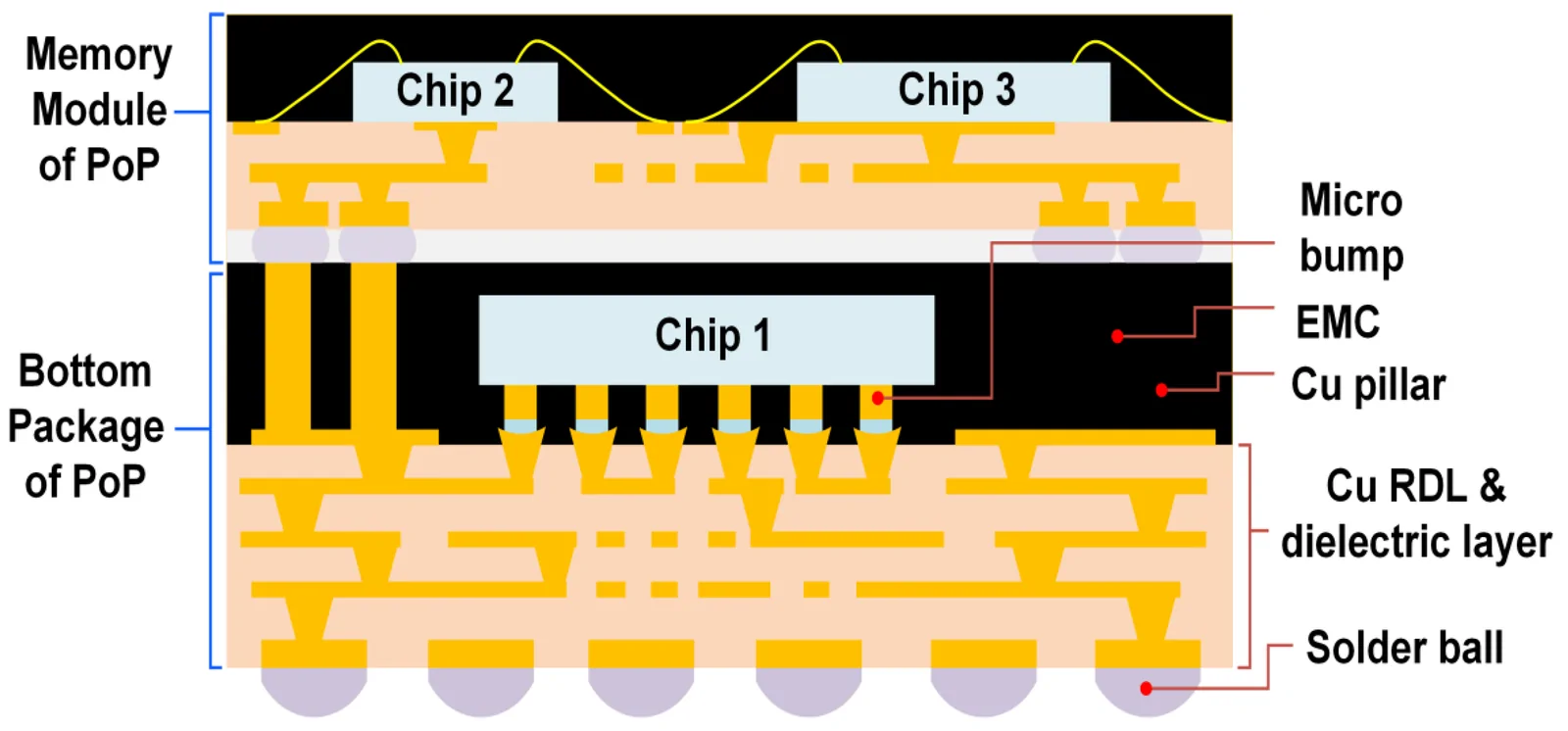
Schematic diagram of the FOPLP packaging structure Adapted from Wang et al. [7].

Fan-Out Panel-Level Packaging (FOPLP) is a further evolution of FOWLP, with its defining feature being the extension of the packaging process scale from wafers to panels. As illustrated in Figure 4, by adopting large-area panel-based manufacturing, FOPLP significantly improves production efficiency and reduces the unit packaging cost while retaining the structural advantages of fan-out packaging.

Depending on the process flow, FOPLP can be classified into two approaches: chip-first and RDL-first. In the chip-first process, known good dies are first placed onto the panel, followed by molding and RDL fabrication, whereas in the RDL-first process, the RDL is formed on the panel prior to chip attachment. Both approaches enable large-scale I/O expansion through the use of RDL, making FOPLP an important packaging solution for meeting the demands of high pin count and high integration density.

### 2.2. Two-And-a-Half-Dimensional (2.5D) System-in-Package (SiP)

#### 2.2.1. Full Silicon Interposers and TSV-Based Platforms

Full silicon-interposer platforms place multiple dies on a lithographically patterned passive or active interposer. Through-silicon vias (TSVs) provide vertical connections to the package substrate, while fine-pitch microbumps connect logic and high-bandwidth memory (HBM) to the interposer. This architecture offers dense and regular routing across a large integration area and is therefore well matched to HPC and AI systems that require wide logic-to-memory interfaces. CoWoS-S is a representative commercial implementation [16,17]. Its principal constraints are interposer area, TSV processing, assembly yield, warpage, and the thermal interaction among adjacent high-power dies. Reported pitch, bandwidth density, and energy per bit depend on the die-to-die interface and product generation and should not be compared without those conditions.

**Figure 5:**
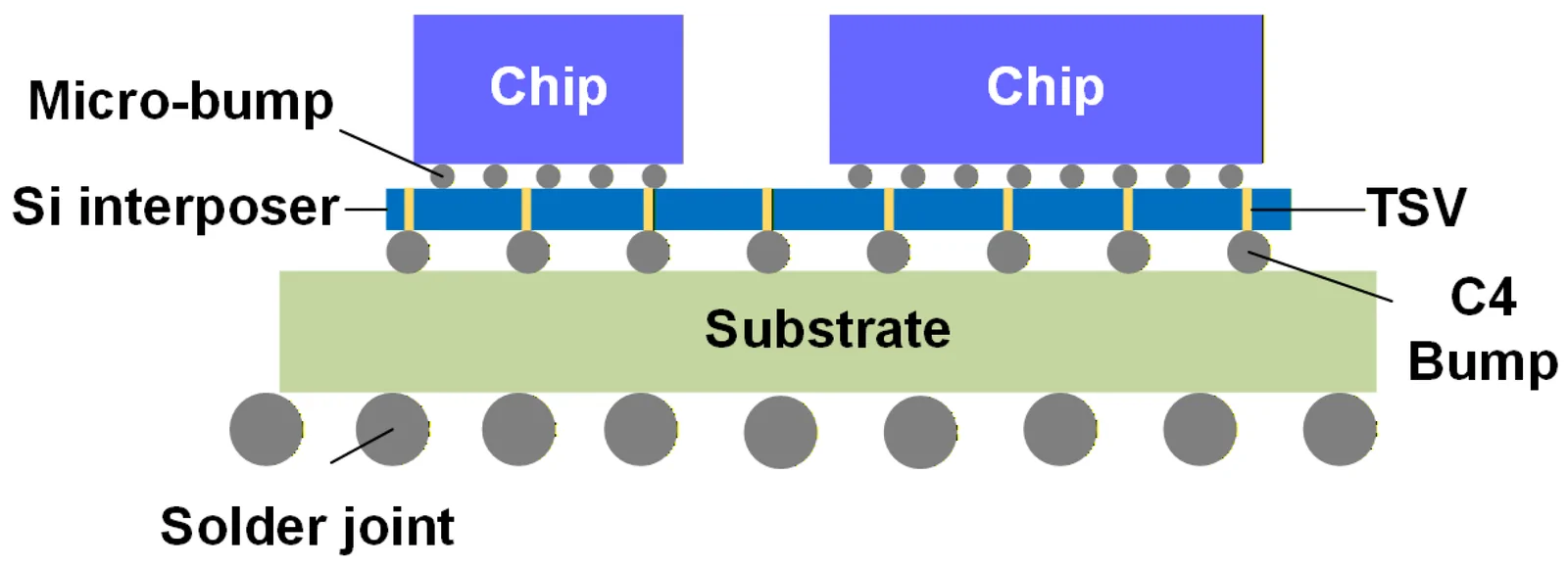
Schematic diagram of a 2.5D packaging structure Adapted from Wang et al. [7].

As shown in Figure 5,the full-interposer route is justified when global routing density and HBM integration dominate cost. It is less attractive when high-density connections are needed only along a few die edges because unused interposer area adds silicon cost and can increase package-level thermo-mechanical complexity. Evaluation should therefore include known-good-die yield, interposer defect density, package size, junction-to-case thermal resistance, microbump fatigue, and total assembled yield rather than interconnect density alone.

#### 2.2.2. Localized Silicon Bridges

A localized silicon bridge provides fine-pitch routing only where adjacent dies require dense communication. Intel’s Embedded Multi-die Interconnect Bridge (EMIB) is a prominent implementation, not a synonym for the entire bridge category. The bridge is embedded in an organic substrate and connected to neighboring dies through localized microbumps, avoiding a package-wide silicon interposer [18,19,20]. IBM’s directly bonded heterogeneous integration (DBHi) uses surface or directly bonded bridge assemblies, including a reported 30-μm-pitch solder-joint test vehicle, while TSMC’s CoWoS-L combines an RDL interposer with embedded local silicon interconnects (LSI) [21,22,23]. These approaches share the objective of concentrating silicon-grade routing near die boundaries but differ in bridge placement, bonding sequence, vertical connectivity, and manufacturing ecosystem.

The bridge approach can reduce silicon area and avoid the TSV array of a full passive interposer, but it transfers difficulty to bridge placement accuracy, substrate cavity formation, coplanarity, local underfill flow, and fine-pitch joint reliability. Multiple bridges may expand the package beyond a reticle-sized interposer, although routing between nonadjacent dies still relies on the organic substrate or additional bridges. Consequently, bridge solutions should be evaluated using edge bandwidth density, bridge-to-die pitch, placement tolerance, local thermal gradients, warpage, repairability, and assembly yield.

Commercial evidence also indicates that architecture names should not be used as direct performance rankings. The baseline comparison is summarized in Table 1, and the common comparison framework is provided in Table 2. EMIB has been deployed in FPGA, processor, accelerator, and HBM-containing products; CoWoS-L targets large HPC integration using RDL plus LSI; and DBHi research emphasizes scalable die tiling. Published pitch values describe different interface locations and qualification states. Table 3 therefore reports ranges or platform-dependent status and retains the original source condition instead of combining values into a single league table.

Dominant reliability concerns include microjoint fatigue, bridge-edge stress concentration, underfill voiding, substrate warpage, and local power-delivery impedance. Removing a full interposer does not remove thermo-mechanical risk; it changes the location and spatial distribution of that risk. Qualification should combine thermal cycling, highly accelerated stress testing, warpage measurement, cross-sectional inspection, and electrical continuity monitoring under representative current and temperature conditions.

**Figure 6:**
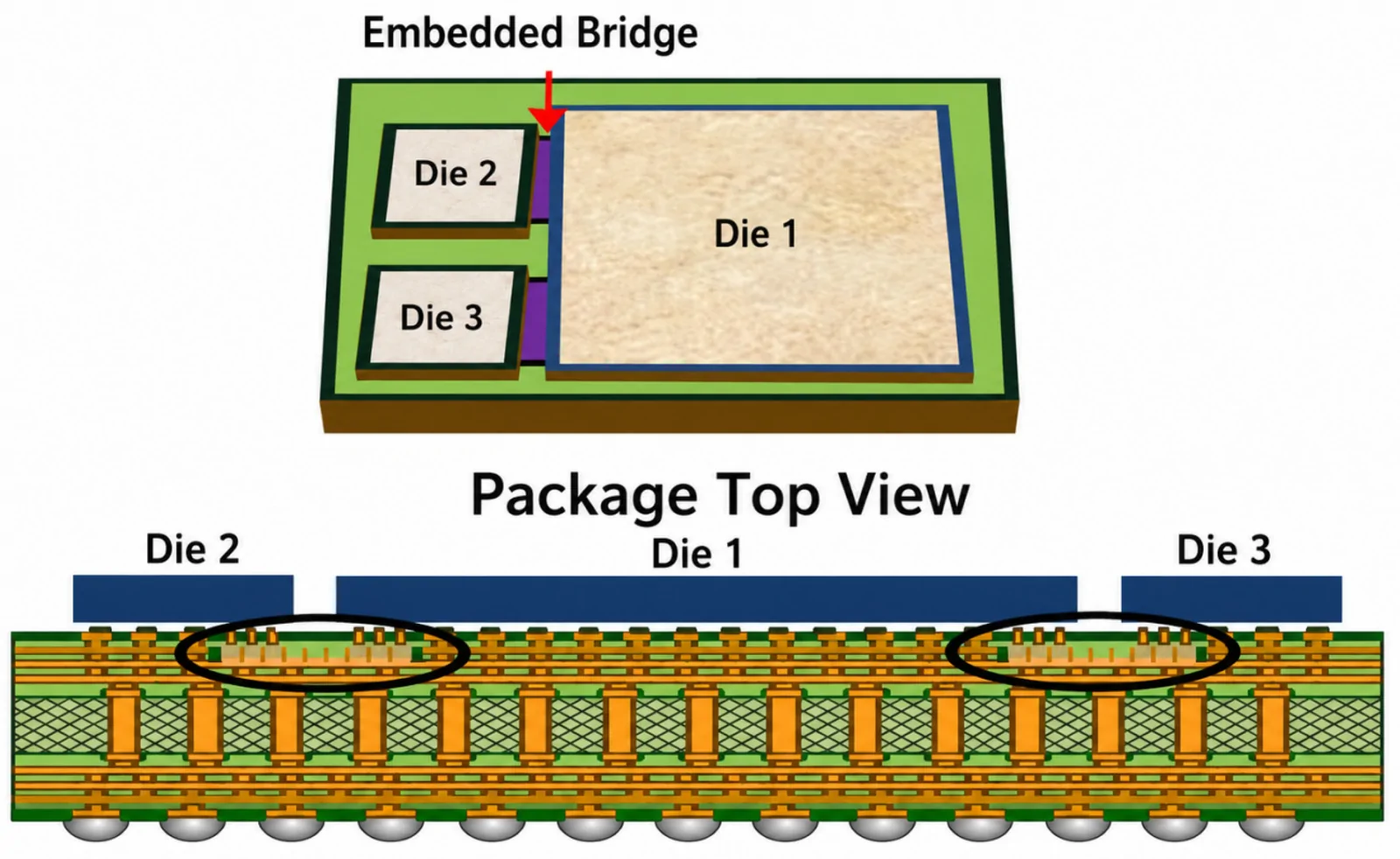
Top view and cross-sectional view of EMIB packaging technology Adapted from Wang et al. [7].

The measurable development targets for localized bridges are shown in Figure 6 and include a finer qualified bridge-to-die pitch, higher edge bandwidth per millimeter at lower energy per bit, a larger multi-bridge package span, improved placement yield, and integrated vertical power and decoupling. These targets must be reported together because an interconnect-density gain that reduces assembly yield or worsens power and thermal margins is not a system-level scaling benefit.

#### 2.2.3. RDL Fan-Out and Bridge-Based Platforms

**Figure 7:**
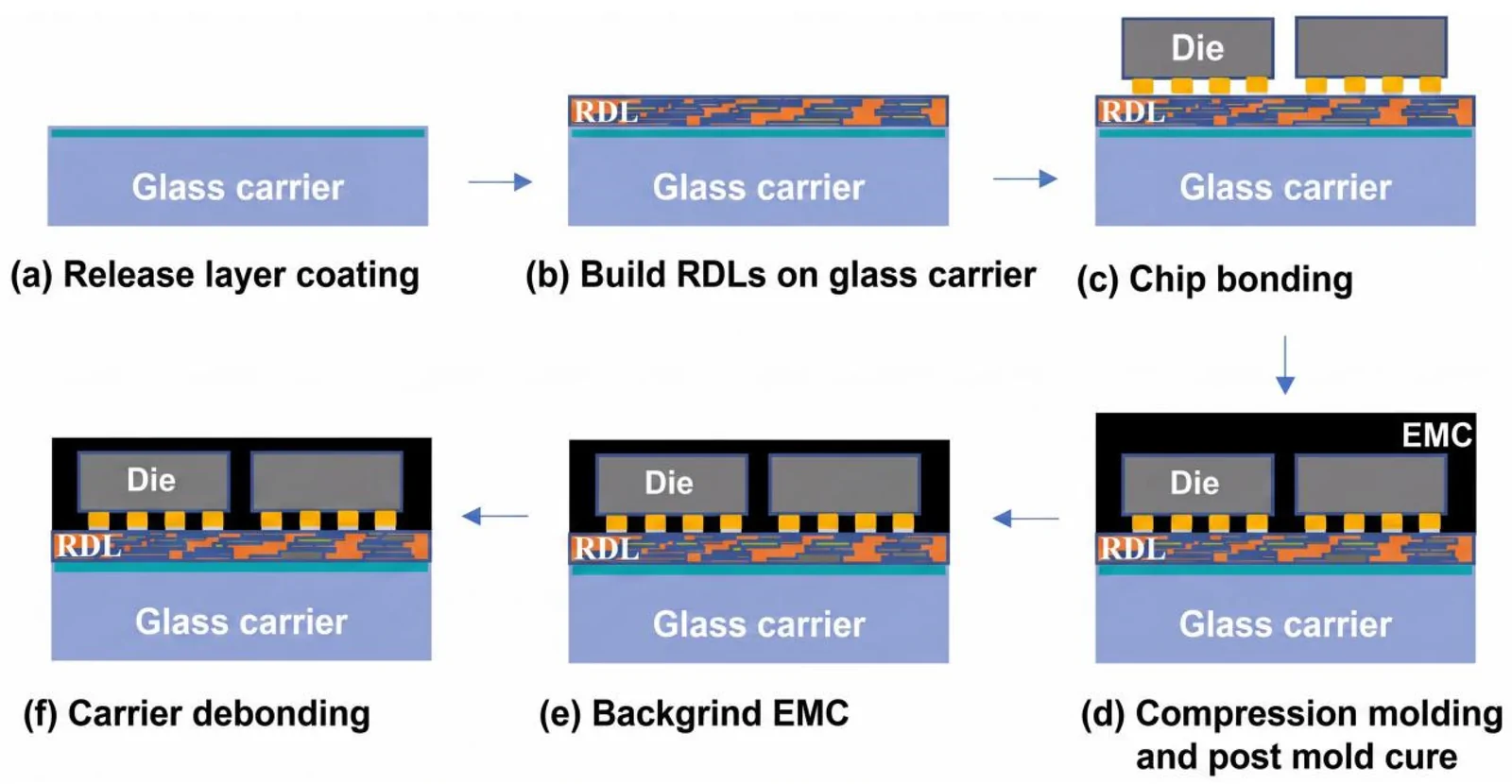
Process flow of Integrated Fan-Out (InFO) packaging Reproduced from Zhao et al. [28].

As shown in Figure 7, Integrated Fan-Out (InFO) is an RDL-centered platform family rather than a localized silicon-bridge architecture. Fan-out processing redistributes die I/O through molded or reconstructed-wafer RDLs and can be implemented in chip-first or RDL-first flows. Some later platforms combine fan-out RDLs with embedded bridges, but the RDL, molding process, and optional bridge must be identified separately. This distinction prevents InFO, EMIB, CoWoS, and SoIC—respectively, a platform family, a bridge implementation, an interposer platform family, and a 3D integration platform—from being treated as equivalent taxonomy levels.

Fan-out routes can achieve thin packages and short signal paths without a full silicon interposer. Their manufacturability is governed by die placement accuracy, mold shrinkage, RDL overlay, panel or wafer warpage, redistribution-layer cracking, and yield across large reconstructed areas [29]. Panel-level processing offers a potential cost advantage, but substrate format, equipment utilization, and yield learning must be reported before wafer- and panel-level costs can be compared.

For mobile and compact heterogeneous systems, the principal benefits are package thickness, integration of passives and multiple dies, and reduced board-level interconnect length. For large HPC packages, RDL fan-out can be combined with a substrate and local bridges to increase integration area. The associated limits are current delivery through thin redistribution structures, thermal spreading through mold compounds, RDL electromigration, and warpage during assembly.

Accordingly, fan-out platforms should be compared by minimum qualified RDL line/space, die-placement tolerance, package or panel size, layer count, warpage, thermal resistance, current-carrying capability, and assembled yield. These conditions determine whether an apparent routing-density advantage remains useful at system scale.

### 2.3. Three-Dimensional (3D) System-in-Package (SiP)

**Figure 8:**
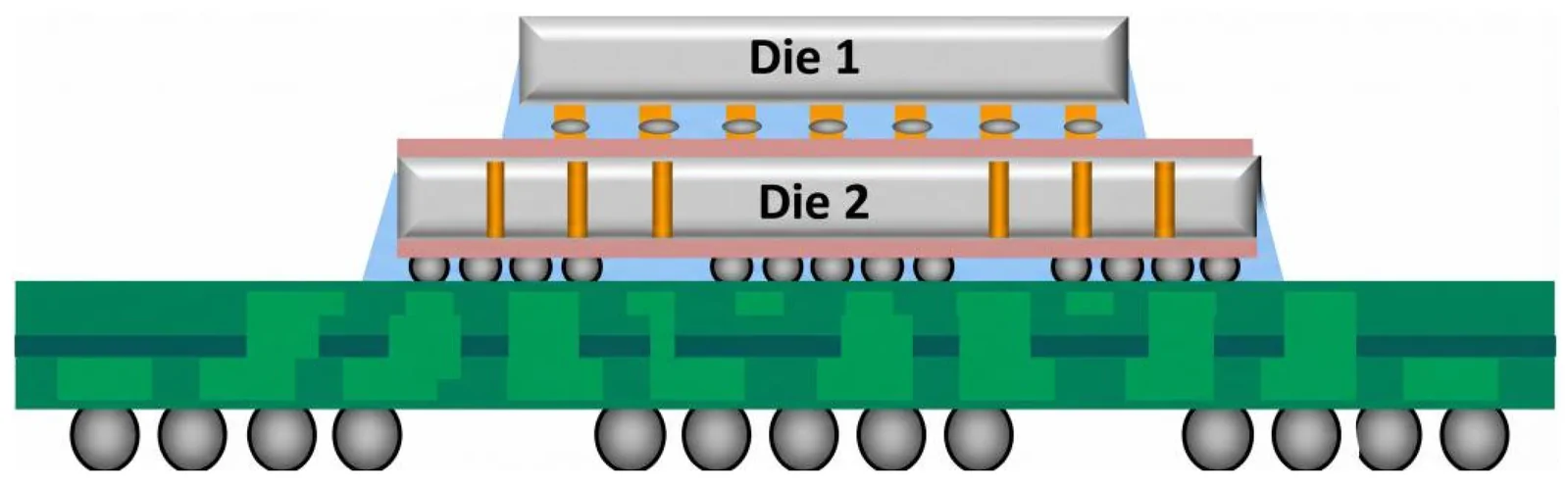
Schematic diagram of 3D packaging structure Adapted from Wang et al. [7].

Three-dimensional (3D) packaging technology achieves the highest level of system integration and the shortest interconnection paths by stacking multiple chips along the vertical direction. As shown in Figure 8, 3D System-in-Package (SiP) structures enable significantly higher functional integration within a limited footprint, making them well suited for applications that are highly sensitive to bandwidth and latency, such as artificial intelligence, high-speed communication, and high-performance computing systems.

#### 2.3.1. Double-Sided Molded Ball Grid Array (DSMBGA)

**Figure 9:**
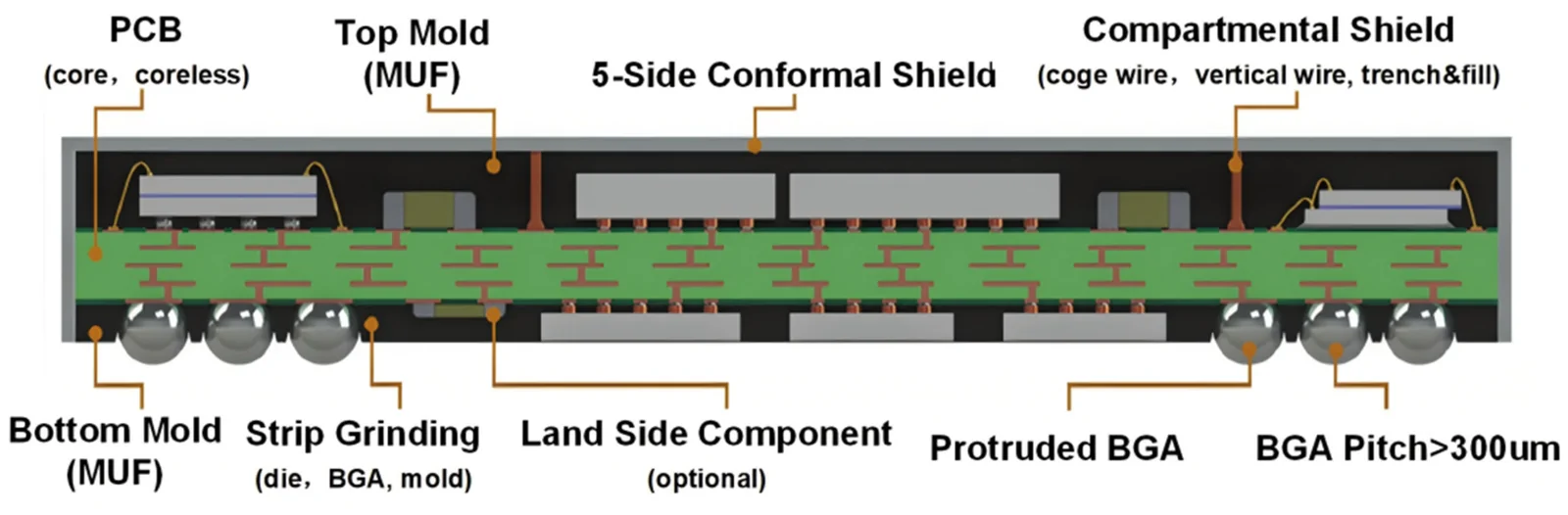
Schematic diagram of the Double-Sided Molded Ball Grid Array (DSMBGA) packaging concept Adapted from Wang et al. [7].

Double-Sided Molded Ball Grid Array (DSMBGA) is a three-dimensional System-in-Package (SiP) technology designed for highly integrated radio-frequency (RF) systems. As illustrated in Figure 9, DSMBGA employs an organic substrate as the core carrier and integrates devices and molded packaging structures on both its top and bottom sides, thereby significantly enhancing system integration within a limited package volume. This packaging format demonstrates particularly outstanding performance in RF front-end modules of mobile terminals such as smartphones [30].

From a structural perspective, DSMBGA extends conventional single-sided SiP packaging by placing active and passive devices on both surfaces of an organic substrate. As shown in Figure 9, the devices are encapsulated by top and bottom molded-underfill (MUF) regions. This double-sided arrangement uses the vertical package volume more efficiently than a planar printed circuit board (PCB) assembly, but it also requires control of mold flow, component clearance, substrate warpage, and access for inspection and test.

For 5G radio-frequency front-end modules, DSMBGA provides a more favorable balance among size, performance, and system complexity. As shown in Figure 9, this structure not only supports the integration of key RF devices such as power amplifiers and filters within the package but also enables the further incorporation of antenna tuners or ultra-wideband (UWB)-related circuits, allowing highly complex RF systems to be realized in a more compact form factor.

At the electrical and reliability levels, DSMBGA also exhibits significant advantages. First, because the devices are encapsulated as an integrated molded structure, the mechanical stress borne by solder joints is substantially reduced compared with implementations where discrete components are soldered onto a PCB, thereby improving interconnection reliability. Second, the protruded ball grid array (BGA) structure shown in Figure 9, together with a relatively large solder ball pitch (BGA pitch > 300 μm), is conducive to improving soldering consistency and enhancing thermo-mechanical reliability.

In addition, DSMBGA packaging exhibits outstanding structural advantages in electromagnetic interference (EMI) suppression. As shown in Figure 9, five-side conformal shielding and compartmental shielding structures can be integrated within the package. Conformal shielding is typically realized by depositing a thin metallic layer on the outer surface of the package via physical vapor deposition (PVD), whereas compartmental shielding constructs localized electromagnetic isolation regions inside the package using approaches such as copper wire grids, vertical conductors, or trench filling. This multi-level EMI/RFI shielding strategy effectively suppresses crosstalk and radiation leakage within RF modules, thereby maintaining system signal integrity.

Overall, DSMBGA achieves a well-balanced trade-off among high integration density, signal integrity, and system reliability through double-sided device integration, integrated molded encapsulation, and multi-level electromagnetic shielding structures, making it one of the key packaging solutions for next-generation high-performance RF SiP modules.

#### 2.3.2. Systems-on-Integrated-Chiplets (SoIC)

**Figure 10:**
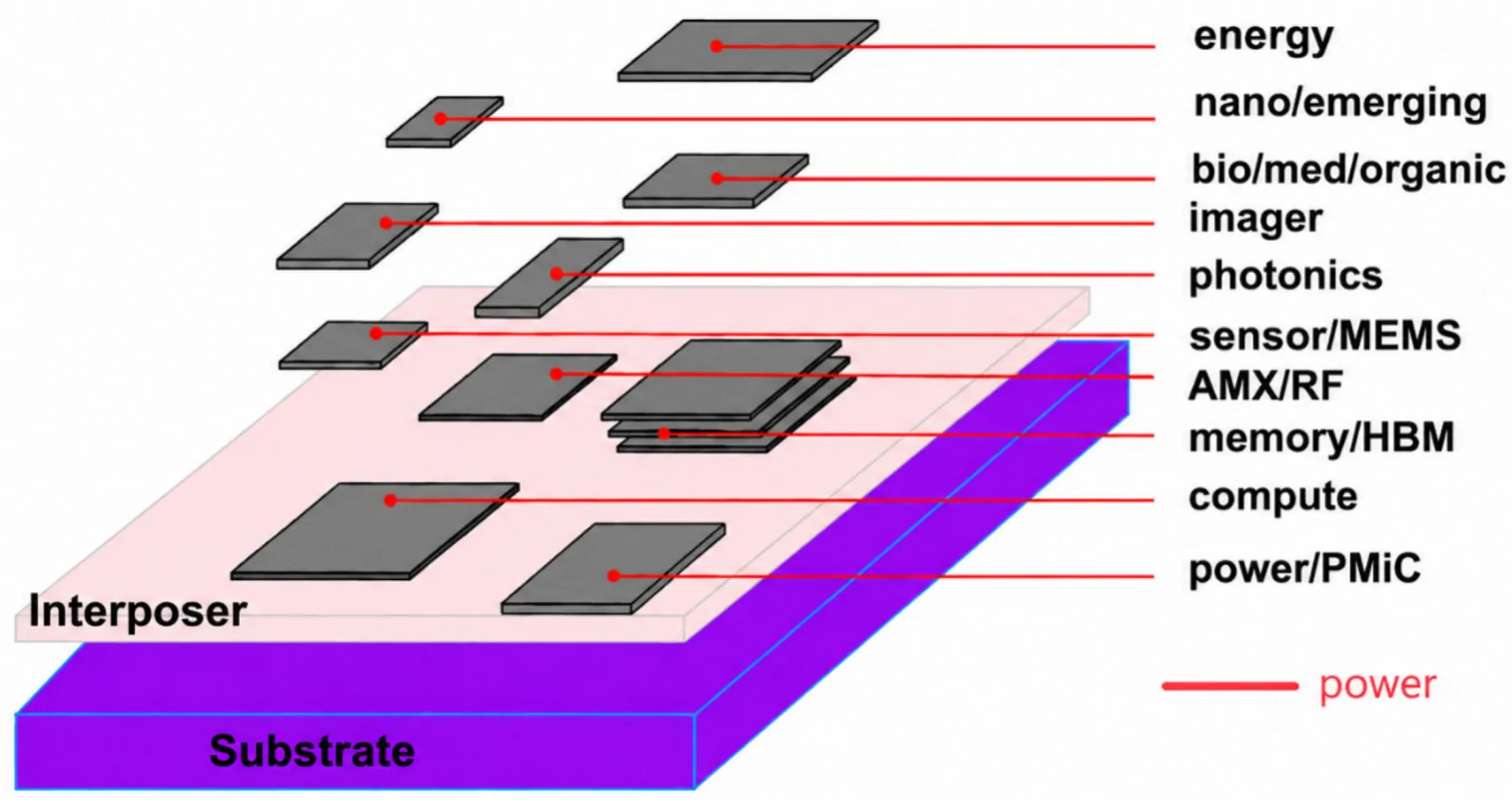
Schematic diagram of Systems-on-Integrated-Chiplets (SoIC) structure Adapted from Li et al. [30].

Systems-on-Integrated-Chiplets (SoIC) is an advanced three-dimensional heterogeneous integration packaging paradigm for the post–Moore’s Law era, which enables system-level functional reconfiguration at the packaging level through heterogeneous integration (HI) and micro-packaging (μ-packaging) technologies. As illustrated in Figure 10, SoIC does not simply stack single-function chips; instead, it uses interposers and substrates as carriers to organically integrate multiple chiplets or dielets with diverse functionalities into a highly cooperative “super chip.”

From a structural perspective, Figure 10 clearly illustrates the multi-functional domain integration characteristics of SoIC. Different functional chips—including compute units, high-bandwidth memory (HBM), analog/RF modules (AMX/RF), sensors/MEMS, photonics, and power management modules (power/PMIC)—are uniformly integrated on a single interposer and tightly coordinated through high-density interconnections. This architecture endows SoIC with system-level functional scalability far exceeding that of conventional single-chip system-on-chip (SoC) solutions.

The realization of SoIC relies on advanced three-dimensional micro-packaging (3D μ-packaging) technologies, whose core characteristic lies in scaling critical packaging interconnect dimensions—such as interconnect pitch and joint size—down to the micrometer or even nanometer level, fundamentally distinguishing it from conventional board-level or module-level packaging. To achieve these objectives, SoIC typically integrates multiple advanced packaging and interconnection technologies, including 2D/2.5D/3D packaging architectures, silicon interposers, silicon bridges, micro-bumps, and copper-to-copper (Cu–Cu) direct bonding [31].

As illustrated in Figure 10, the interposer plays a critical role in the SoIC architecture, serving not only as a high-density signal interconnection platform but also as the electrical and physical interface between chips fabricated with different process nodes and material systems. Representative three-dimensional micro-packaging implementations include Chip-on-Wafer-on-Substrate (CoWoS) technology, which has been successfully deployed in high-performance computing and artificial intelligence accelerators.

## 3. Applications of Advanced Packaging Technologies

Application value is determined by whether a package resolves a specific system bottleneck. The relevant sequence is therefore a requirement, selected architecture, measurable benefit, and residual limitation. Table 4 summarizes this selection logic. The following subsections retain representative use cases but avoid treating application growth alone as evidence that a packaging approach is suitable.

### 3.1. Power Module

**Figure 11:**
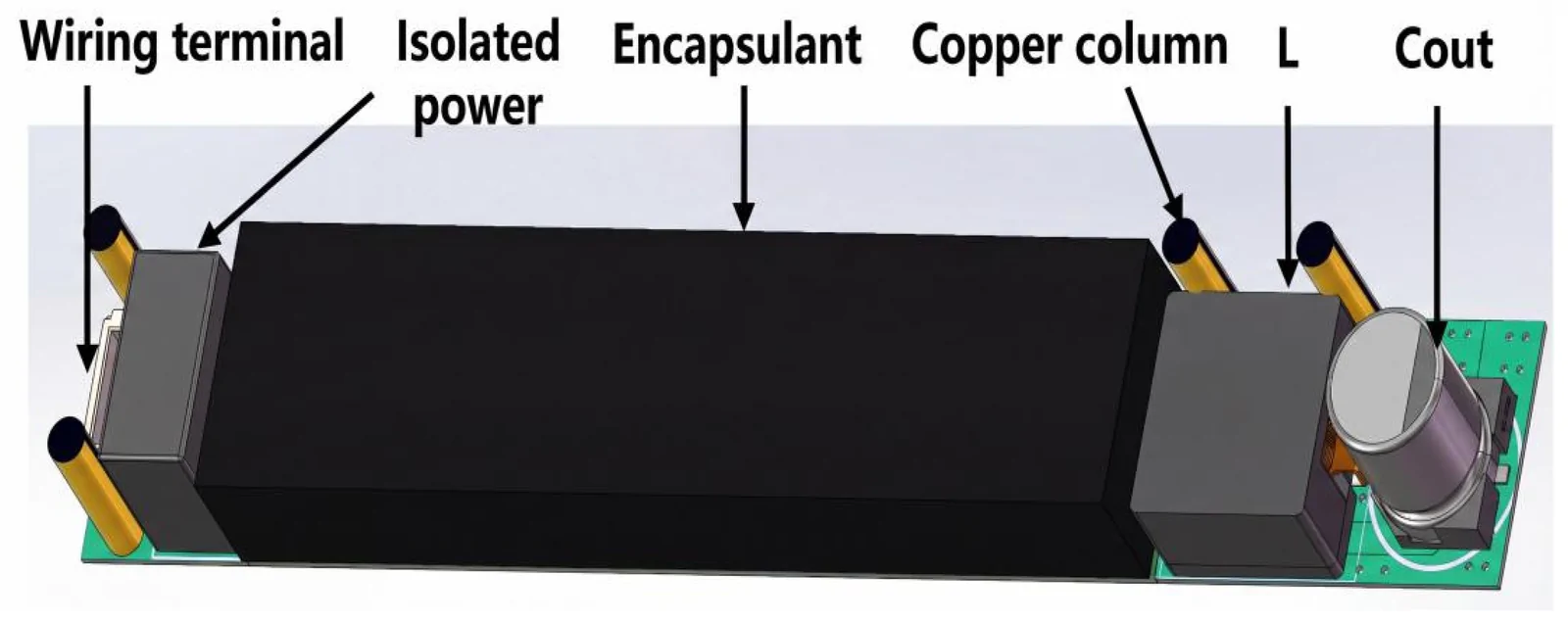

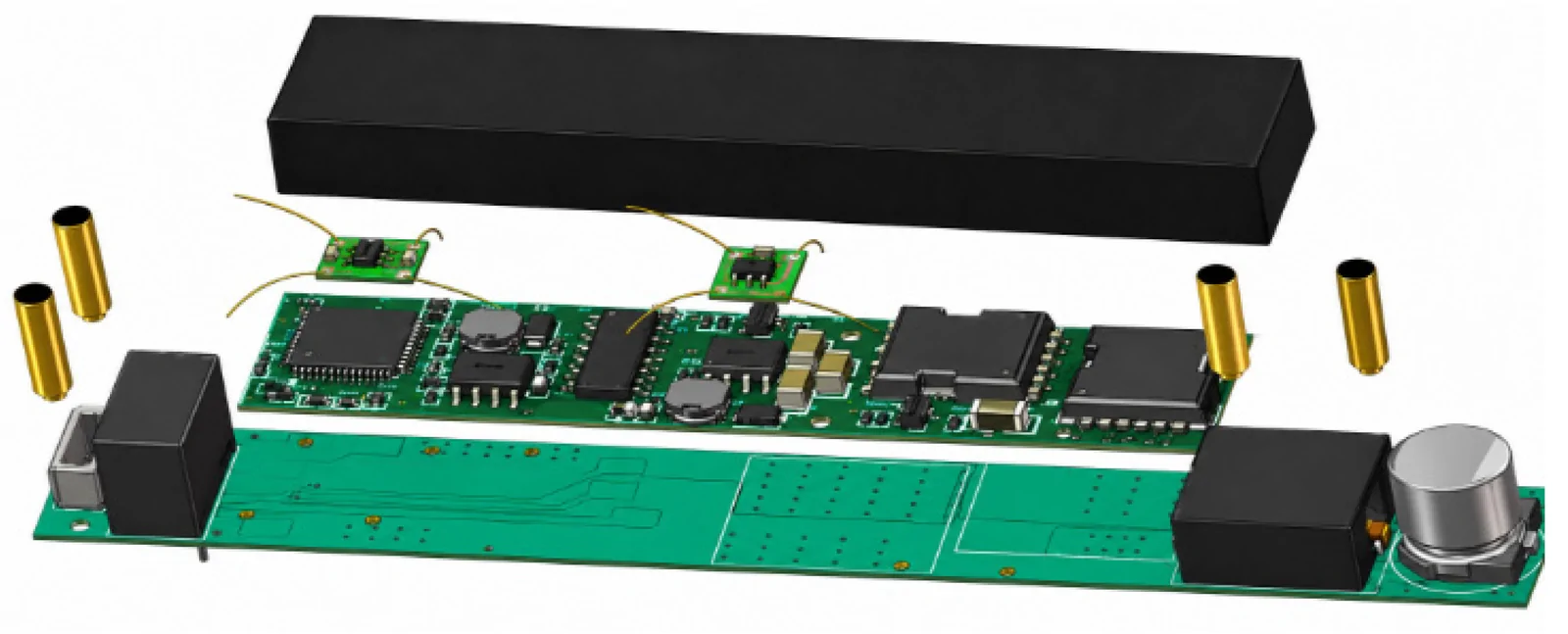
Schematic diagram of power module advanced packaging structure.

Power modules are core components of various power electronic systems, whose development has long been constrained by the trade-offs among power density, thermal management, and reliability. Advanced packaging technologies, particularly System-in-Package (SiP) and multidimensional integration architectures, provide effective pathways to resolve the conflict between high power capability and miniaturization. As shown in Figure 11, advanced packaging technologies have been widely applied in high–power-density power modules.

In application scenarios such as automotive and aerospace systems, power modules often operate under high-temperature and severe vibration environments, imposing stringent requirements on the mechanical stability and thermal reliability of packaging. By integrating power devices, driver chips, and passive components into a single module through three-dimensional packaging structures, and by optimizing solder ball structures and layouts, stress distribution can be effectively improved and system reliability enhanced [33]. In terms of thermal management, to address the heat dissipation challenges associated with the high power density of SiC devices, advanced packaging solutions such as double-sided cooling (DSC) significantly reduce junction temperature and thermal resistance by establishing heat dissipation paths on both the top and bottom sides of the devices [34,35].

In addition, advanced packaging technologies also exhibit significant advantages in electrical performance optimization. By adopting leadless interconnection schemes, optimizing current loop layouts, and leveraging mutual inductance cancellation effects, parasitic inductance can be substantially reduced, thereby mitigating voltage overshoot and decreasing switching losses [36,37]. With the aid of 2.5D or multilayer integration approaches, power modules are able to achieve high power density while simultaneously satisfying requirements for efficiency, form factor, and reliability [38].

### 3.2. High-Performance Computing (HPC) and Artificial Intelligence (AI)

**Figure 12:**
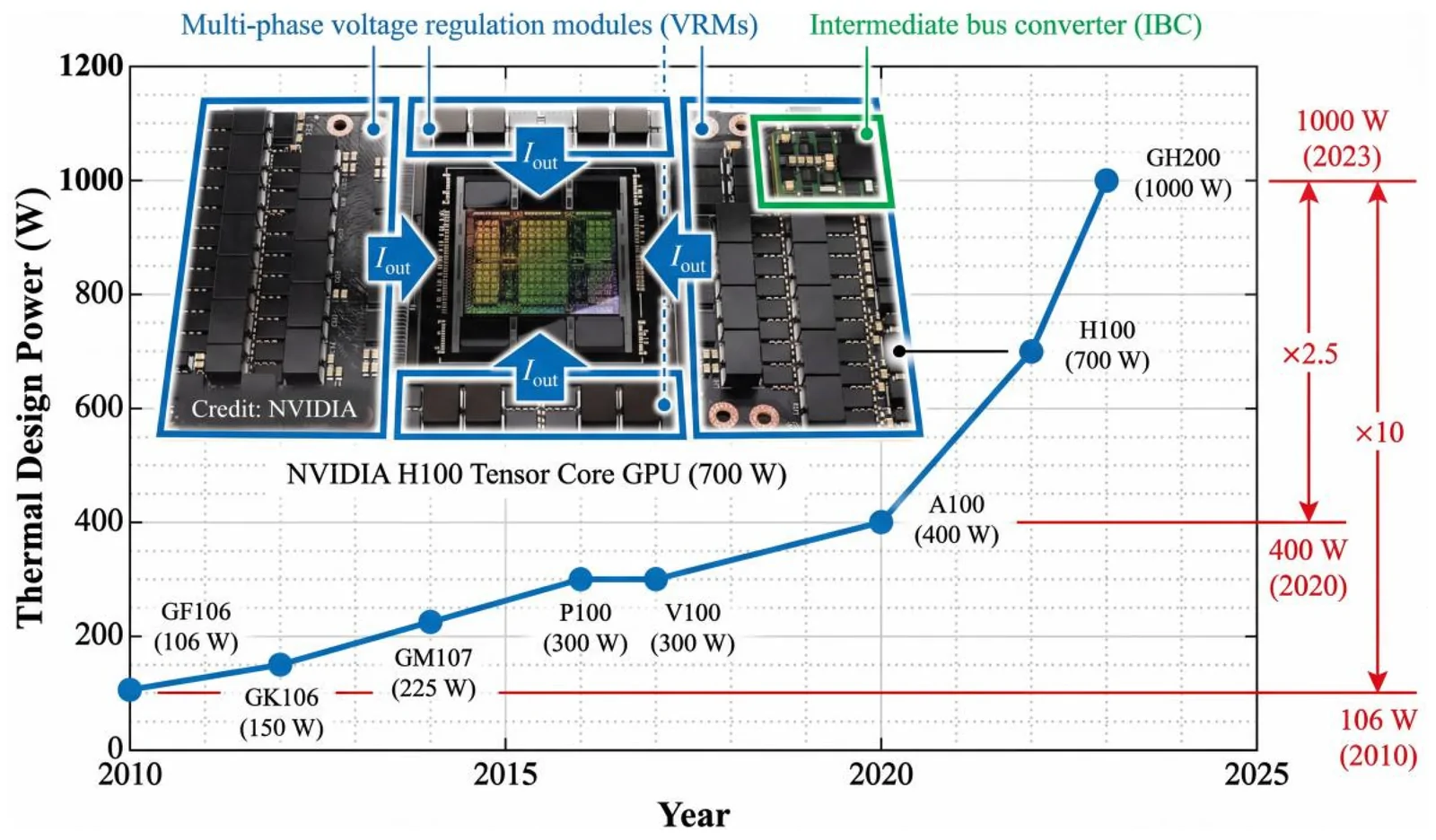
Rising power density in AI and high-performance computing chips and the corresponding need for advanced electronic packaging that co-optimizes power delivery, thermal management, and heterogeneous integration at the package level. Compiled from publicly reported device specifications and refs. [16,17,18,19,20,39,40].

With the rapid growth of artificial intelligence (AI) and data center computing demands, the thermal design power (TDP) of processors has exhibited a pronounced generational scaling trend. As illustrated in the Figure 12, from early GPUs with power consumption on the order of hundreds of watts to recent devices such as H100 and GH200 reaching several hundred watts or even kilowatt-level power, single-chip computing capability and power density have continued to increase. This trend not only imposes more stringent requirements on the chips themselves, but also causes conventional packaging as well as board-level power delivery and interconnection solutions to increasingly become bottlenecks for further system performance improvement.

In this context, advanced packaging technologies—particularly 2.5D and 3D integrated packaging—have become key enablers for next-generation high-performance computing (HPC) and AI accelerators. Through 2.5D packaging, multiple functional chips are integrated side by side on a silicon interposer, and high-density micro-bumps are used to realize high-speed interconnections among GPUs, CPUs, and high-bandwidth memory (HBM), thereby significantly increasing system bandwidth and reducing communication latency [39]. This architecture effectively aligns with the core requirements of AI workloads for high computing capability, high bandwidth, and low latency.

However, with the simultaneous increase in chip power consumption and integration density, 2.5D packaging is also facing increasingly prominent thermal and mechanical reliability challenges. Under high power-density operating conditions, localized hotspot issues become more pronounced, and the power escalation trend shown in the figure directly amplifies the risk of thermally induced stress within the package. To address these issues, researchers have introduced high-thermal-conductivity heat spreaders, optimized thermal interface materials (TIMs), and adjusted chip thickness and layout to achieve more uniform heat dissipation paths, thereby reducing thermal resistance and alleviating thermal stress concentration.

In terms of specific technological implementations, TSMC’s CoWoS (Chip-on-Wafer-on-Substrate) technology, as a representative 2.5D packaging solution, has been widely adopted for high-bandwidth integration of multi-core processors and HBM to meet the extreme memory bandwidth demands of AI systems [16,17]. In contrast, Intel’s EMIB technology achieves high-density interconnections through localized silicon bridges, striking a balance among performance, cost, and scalability while avoiding the use of large-area silicon interposers [18,19,20]. In addition, to address future demands for higher I/O density and lower power consumption, research on ultra-low dielectric-constant and ultra-thin polymer dielectric materials is actively progressing, providing a material foundation for further enhancing the electrical performance of package interconnections [40].

Overall, the trends in power consumption and computing capability growth shown in the figure clearly indicate that advanced packaging has evolved from a “performance optimization approach” into a “fundamental enabling technology” for HPC and AI systems. While continuously enhancing system performance, achieving system-level co-optimization among thermal management, mechanical reliability, and electrical integrity has become a core challenge that advanced packaging must urgently address in data center and AI accelerator applications.

### 3.3. Radar and Communication

With the rapid development of fifth-generation (5G) communication technology, communication and radar systems have imposed higher requirements on RF front ends, including higher operating frequencies, wider instantaneous bandwidths, higher power efficiency, and more compact system form factors. In millimeter-wave communications and high-frequency radar applications, conventional board-level integration approaches have gradually become performance bottlenecks in terms of signal loss, parasitic effects, and heat dissipation capability. Consequently, advanced packaging technologies have come to play an increasingly important role in wireless communication and radar systems.

Fan-Out Wafer-Level Packaging (FOWLP) effectively shortens high-frequency signal transmission paths by integrating RF chips, power modules, and associated passive components at the packaging level, thereby reducing parasitic inductance and capacitance and improving RF signal integrity. Its planar interconnection structure based on redistribution layers (RDLs) facilitates compact layouts and impedance continuity control, enabling FOWLP to demonstrate strong application potential in 5G RF front-end modules and millimeter-wave communication chips.

In 5G base-station transceivers as well as high-performance radar and communication systems for defense and aerospace applications, gallium nitride (GaN) RF power amplifiers have become one of the key devices due to their high power density, high breakdown voltage, and excellent power-added efficiency [41]. However, under high power-density operating conditions, the substantial heat generated by GaN devices, if not effectively dissipated, will directly limit their output performance and accelerate reliability degradation.

**Figure 13:**
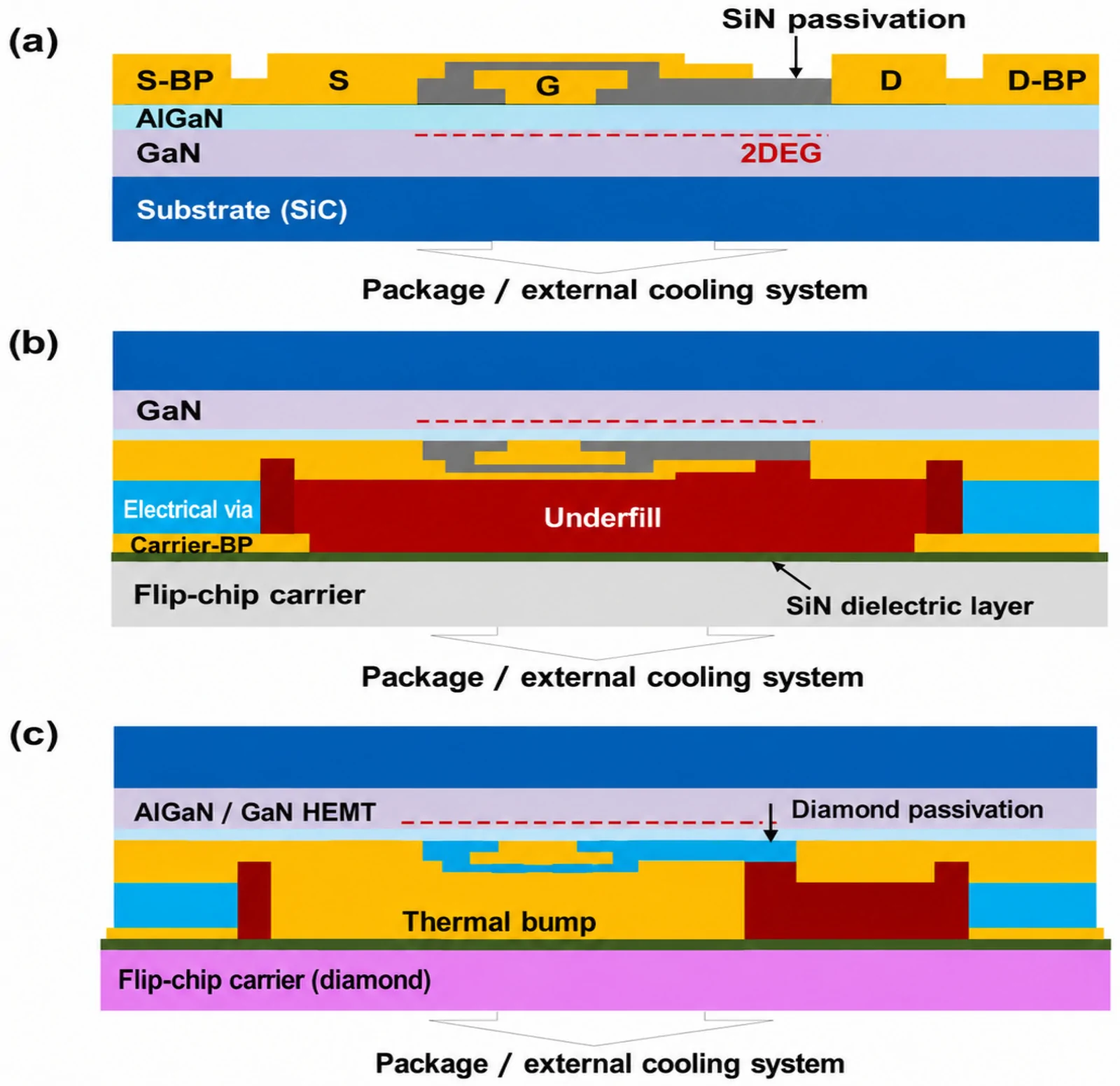
Cross-section of GaN HEMT devices with different packaging and thermal management configurations: (**a**) conventional AlGaN/GaN HEMT structure on a SiC substrate with SiN passivation; (**b**) flip-chip packaged device with electrical vias, underfill, and an external cooling system; (**c**) diamond-integrated flip-chip package with thermal bump and diamond passivation for enhanced heat dissipation. Adapted from Shoemaker, D. et al. [40].

As shown in Figure 13, comparative cross-sectional schematics of GaN HEMT devices with different packaging and thermal management structures highlight the critical role of advanced packaging in heat dissipation. By adopting flip-chip configurations, introducing high-thermal-conductivity thermal bumps, and integrating diamond passivation layers or high-thermal-conductivity carriers on the device surface or carrier side, more direct and efficient heat conduction paths can be established to rapidly transfer the generated heat to the package and external cooling systems. Relevant studies indicate that such co-design of advanced packaging and materials can significantly reduce the junction-to-package thermal resistance and can even achieve thermal resistance levels lower than those of conventional GaN-on-diamond high-electron-mobility transistor (HEMT) structures [26].

By co-optimizing high-frequency interconnections and high-efficiency thermal management strategies at the packaging level, advanced packaging technologies provide critical support for the reliable operation of GaN RF power devices in 5G communication and high-performance radar systems.

In addition, System-in-Package (SiP) technology integrates the RF front end, power management, and other critical components into a single package, effectively reducing electromagnetic interference (EMI) and improving signal quality [42]. In automotive applications, radar-on-chip or radar-in-package solutions have become feasible, primarily enabled by the integration of package-level antenna arrays, multi-channel RF transceivers, and high-performance analog-to-digital converters (ADCs) within system-on-chip (SoC) platforms. These highly integrated solutions significantly reduce the cost and power consumption of automotive radar systems and support advanced signal processing techniques such as MIMO (multiple-input multiple-output) and cognitive radar, thereby playing a critical role in autonomous driving and intelligent transportation systems.

### 3.4. MEMS Sensors

Micro-electromechanical systems (MEMS) sensors have been widely applied in fields such as smart homes, automotive electronics, healthcare, and data centers, with development trends increasingly oriented toward miniaturization, low power consumption, and multifunctional integration. Conventional MEMS devices typically rely on discrete packaging and board-level interconnections, which not only occupy substantial system space but also exhibit inherent limitations in terms of power consumption and reliability. System-in-Package (SiP) technology provides an effective approach to achieving high integration density and low power consumption by integrating MEMS sensors with functional units such as signal processing chips and communication modules within a single package.

MEMS devices themselves feature diverse structures and highly integrated functionalities, encompassing a wide range of sensing and actuation capabilities such as pressure, acceleration, gas detection, and optical modulation. By leveraging advanced packaging technologies such as flip-chip and fan-out packaging, multifunctional co-integration can be achieved at the packaging level, reducing reliance on multiple discrete modules in conventional systems and thereby lowering manufacturing complexity while enhancing overall system reliability.

**Figure 14:**
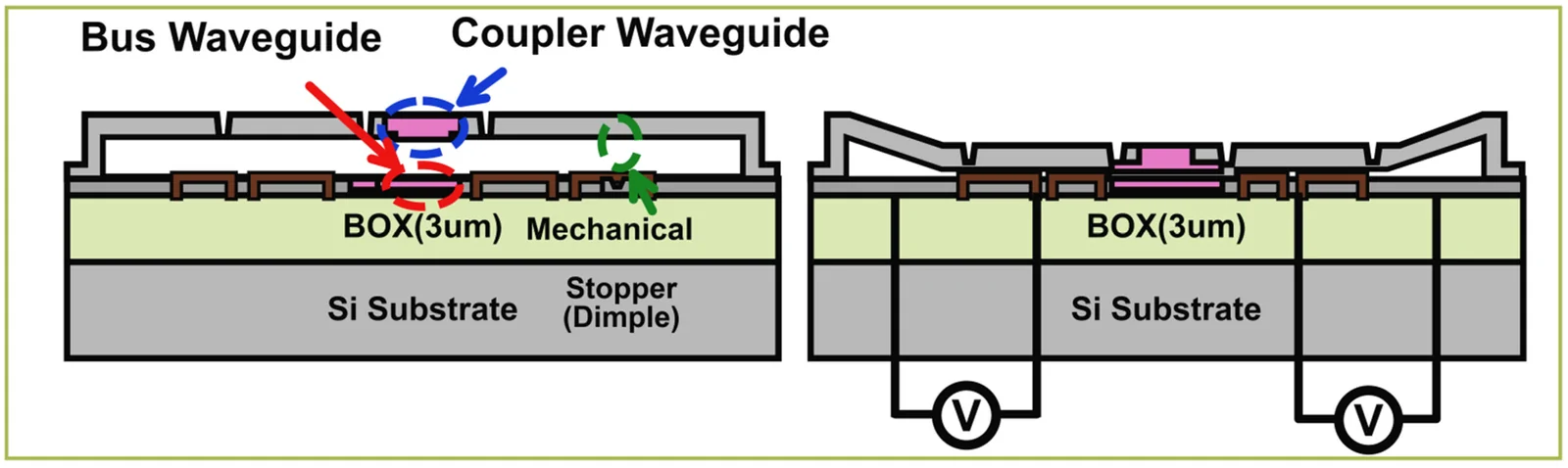
Typical structure of a silicon photonic MEMS switch Adapted from Hwang et al. [27].

As shown in Figure 14, in a typical structure of a silicon photonic MEMS switch, optical waveguides, coupling regions, and MEMS actuation structures are integrated on top of the buried oxide (BOX) layer, and device displacement is precisely controlled by mechanical stoppers to ensure stable optical coupling. Such devices are of significant application value in optical interconnect systems for cloud computing and data centers, where they can be used to realize high-speed routing of large-scale data traffic [41]. For an N × N digital optical switch system, N^2^ electrical interconnections and 2N optical interconnections are typically required, which places extremely stringent demands on packaging in terms of interconnect density, alignment accuracy, and reliability.

Conventional wire-bonding schemes, due to their long interconnect wires, are prone to introducing mechanical sagging and electrical parasitic effects, while the suspended MEMS structures also limit the use of underfill materials. To address these issues, researchers have adopted flip-chip packaging to directly interconnect MEMS optical switch chips onto aluminum nitride (AlN) ceramic interposers, thereby reducing mechanical stress induced by coefficient of thermal expansion (CTE) mismatch and improving overall reliability [26]. In addition, by employing laser solder ball jetting and fluxless reflow soldering processes, residue formation near MEMS actuators can be avoided, ensuring free device movement and enabling high-precision alignment with fiber arrays [42].

Benefiting from the aforementioned co-design of packaging and device structure, silicon photonic MEMS switches achieve sub-microsecond switching speeds, low on-chip optical loss, and wide optical bandwidth while maintaining structural reliability, fully demonstrating the critical enabling role of advanced packaging technologies in MEMS and photonic integration.

### 3.5. Cross-Disciplinary Applications

**Figure 15:**
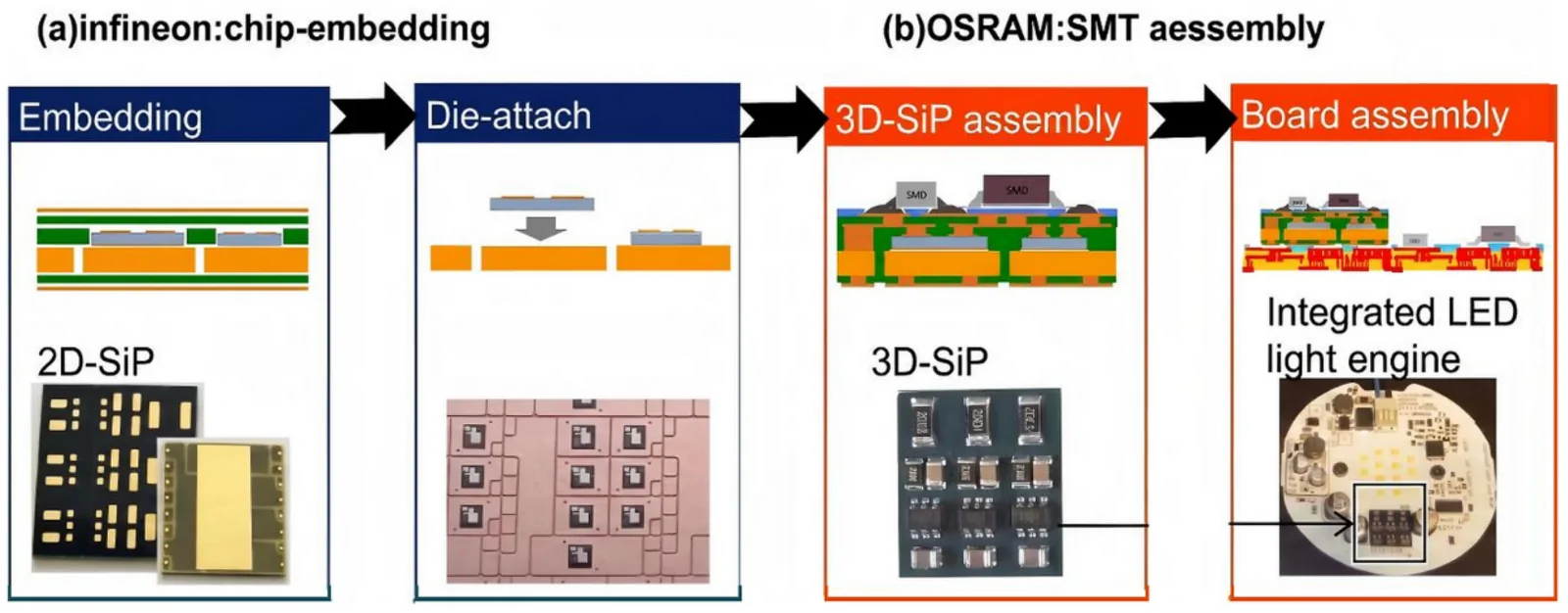
Schematic diagram of the application of advanced packaging in lighting modules Adapted and redrawn from Fruehauf, P. et al. [42].

Beyond traditional domains such as communications, computing, and sensors, advanced packaging technologies have also found application scenarios across multiple interdisciplinary fields. As illustrated in Figure 15, advanced packaging is applied in lighting modules. In areas including photonic chip packaging, laser integration, and automotive electronics, advanced packaging technologies are playing an increasingly important role.

In the field of photonic chips, as the demand for optical communications and optical computing continues to increase, System-in-Package (SiP) technology enables the integration of optical components, electronic devices, and sensors within a single package, thereby enhancing optoelectronic performance and integration density. For example, femtosecond lasers and photonic sensors can achieve more efficient signal transmission and precise control through advanced packaging technologies. In a flip-chip packaging study of a digital silicon photonic micro-electromechanical system (MEMS) switch for cloud computing and data center applications, researchers addressed the challenges associated with large-scale electrical and optical interconnections by employing an aluminum nitride (AlN) interposer [27]. This packaging scheme achieved precise alignment between the device and the interposer through laser solder ball jetting and fluxless reflow soldering, while avoiding interference with the motion of MEMS actuators. Ultimately, the package enabled sub-microsecond switching reconfiguration times, demonstrating the significant potential of advanced packaging technologies in the field of photonic integration.

In the field of automotive electronics, the advancement of autonomous driving technologies has imposed new requirements on intelligent vehicles for electronic systems with high density, high integration, and low power consumption. Advanced packaging technologies enable the integration of multiple electronic modules within in-vehicle systems, improving system responsiveness and reliability while effectively controlling power consumption. For example, in radar chip packaging, System-in-Package (SiP) and System-on-Chip (SoC) technologies allow the integration of multi-channel radio-frequency (RF) transceivers, high-performance analog-to-digital converters (ADCs), and embedded processing platforms, thereby enabling low-cost and low-power radar-on-chip solutions. In addition, to address reliability challenges under harsh operating environments, researchers have developed laminated chip embedding technologies based on copper lead frames (Cu-LF) and conducted reliability validation for packages used in DC/DC converters and lighting applications. The results demonstrate that such packaging solutions can maintain robust performance under stringent conditions such as thermal cycling, humidity exposure, and switching stress tests [43].

## 4. Reliability Analysis and Challenges

With the widespread application of advanced packaging technologies in fields such as high-performance computing, communications, and power electronics, packaging not only enhances system performance and integration density but also introduces increasingly complex reliability issues. Particularly under conditions of high power density, high integration density, and multi-material heterogeneous integration, thermal management, mechanical stress, and electrical reliability have become critical factors constraining the long-term stable operation of advanced packaging technologies [41,42,43,44,45,46,47]. Therefore, it is necessary to systematically analyze the reliability challenges of advanced packaging from a multiphysics coupling perspective.

### 4.1. Thermal Management and Thermal Reliability

#### 4.1.1. Thermal Management Strategies

(1) Use of high-thermal-conductivity materials

The use of high-thermal-conductivity materials is an effective approach to reducing thermal resistance. For example, in 3D integration, through-silicon vias (TSVs) not only serve as electrical interconnects but also function as important heat conduction paths. Studies have shown that replacing conventional copper-filled TSVs with high-thermal-conductivity filling materials such as carbon nanotubes can significantly reduce chip temperature [48,49,50]. In addition, improving the performance of thermal interface materials (TIMs) can effectively lower interfacial thermal resistance [51].

(2) Structural optimization

**Figure 16:**
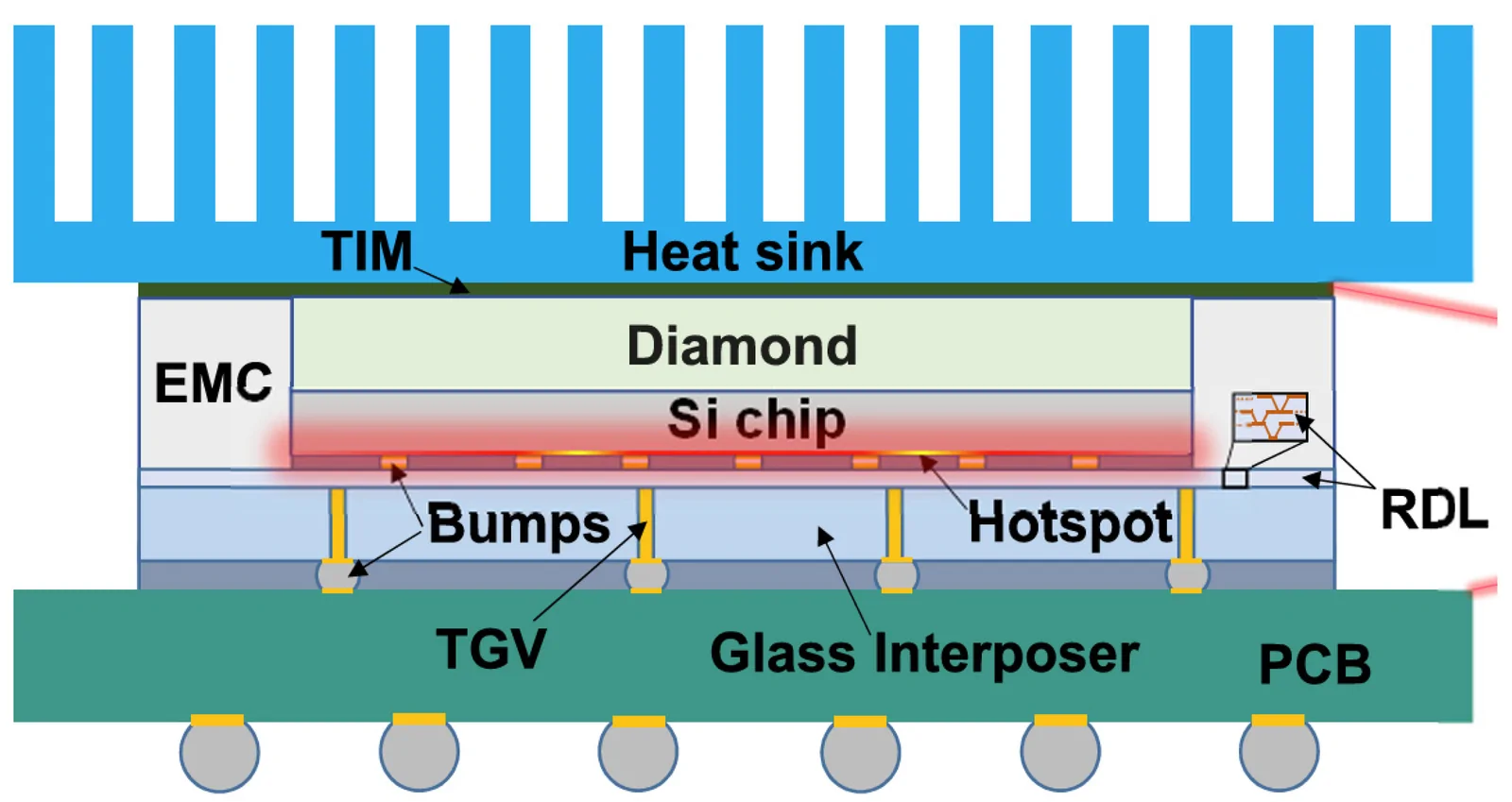
Illustrates the effect of packaging structural optimization on heat dissipation performance in advanced packages. Adapted and redrawn from Zhong et al. [51].

Rational packaging structure design is another key factor in effective thermal management. As shown in Figure 16, in 3D packaging, optimizing the contact area between the chip and the substrate as well as the heat conduction paths can significantly enhance thermal efficiency. For example, researchers have successfully directly bonded polycrystalline diamond heat-spreading substrates to silicon chips on glass interposers, reducing the junction-to-ambient thermal resistance by 28.5% and achieving excellent heat dissipation performance [52]. In addition, increasing the radius of TSVs and reducing their pitch can enhance lateral heat conduction toward the TSVs, thereby lowering the temperature of the chip layers [53,54].

(3) Microfluidic cooling technology

In some high-power and high-density packages, conventional cooling methods are no longer sufficient to meet thermal management requirements. Microfluidic cooling technology introduces microscopic liquid channels within the package to remove heat through fluid flow, representing an effective cooling approach [55]. For example, micro-pin-fin heat sinks (MPFHSs) can be directly etched on the backside of chips, efficiently dissipating heat by increasing the effective surface area and reducing the overall thermal resistance to as low as 0.286 °C·cm^2^/W. This technology has demonstrated significant potential in applications such as high-performance computing (HPC) and data centers [32].

(4) Dynamic thermal management (DTM)

**Figure 17:**
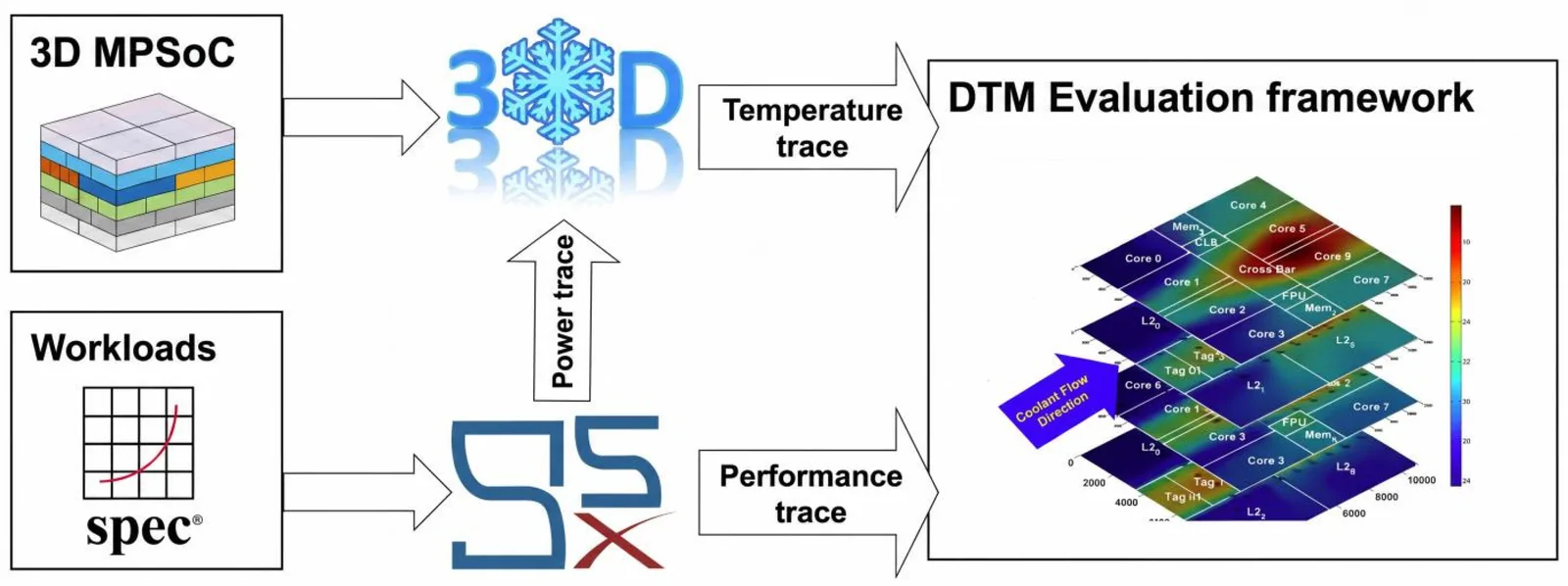
Operating framework of dynamic thermal management based on power–temperature modeling: arrows indicate the flow of workload, power, temperature, and performance traces; the color scale denotes temperature. Reproduced from Huang et al. [55].

For complex systems such as multiprocessor system-on-chip (MPSoC) platforms with pronounced time-varying power consumption and thermal behavior, dynamic thermal management (DTM) has become a key technology for ensuring performance and reliability. As illustrated in Figure 17, DTM typically maps runtime power consumption to temperature evolution based on workload characteristics and power profiles through power and thermal modeling, and establishes a unified evaluation and decision-making framework on this basis. During system operation, DTM continuously monitors the temperature and performance states of individual processing cores and suppresses localized hotspots through dynamic voltage and frequency scaling (DVFS), thereby effectively controlling peak temperatures without fully shutting down cores. This strategy reduces thermal stress and device aging risks while meeting performance requirements, enabling a dynamic trade-off among power consumption, performance, and reliability, and is particularly suitable for applications such as three-dimensionally stacked MPSoCs with strong thermal coupling and highly variable workloads [56].

#### 4.1.2. Thermal Simulation and Testing

**Figure 18:**
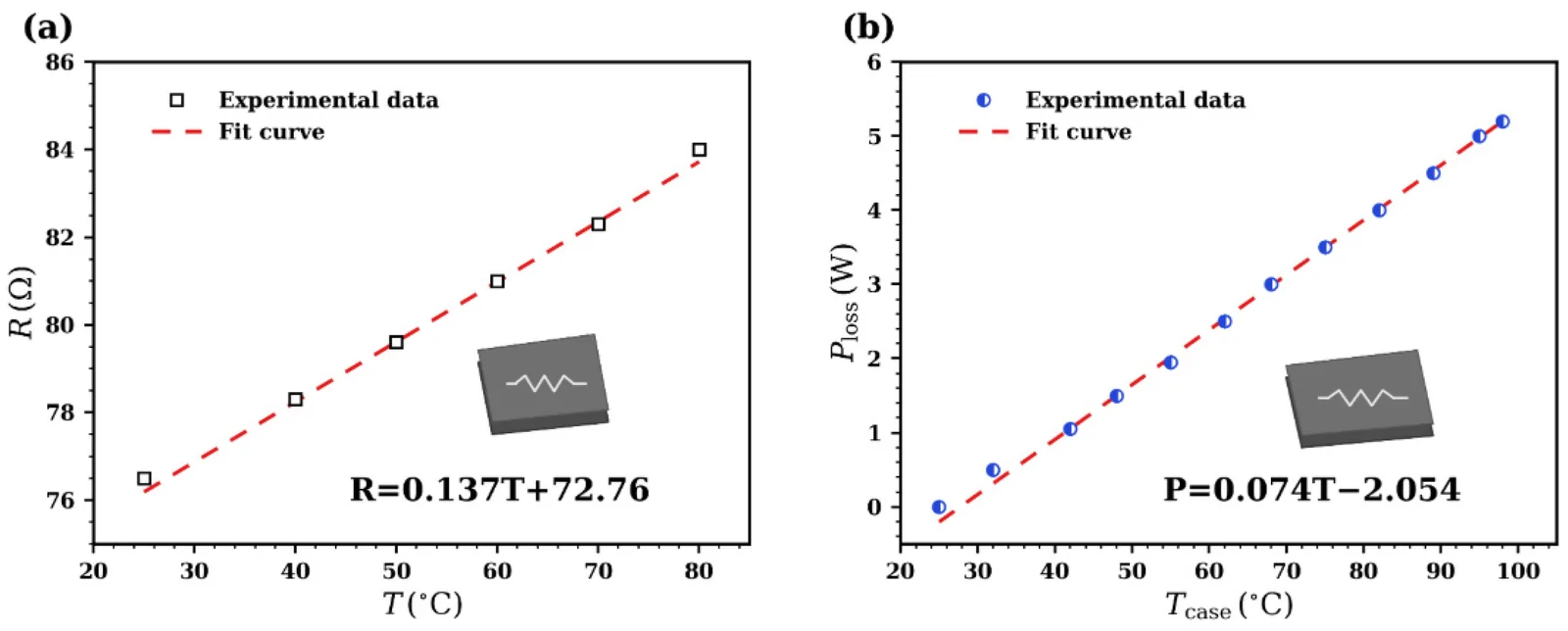
Thermal testing of the chip: (**a**) measured resistance as a function of temperature and the corresponding linear fit; (**b**) measured power loss as a function of case temperature and the corresponding linear fit. Reproduced from Xu et al. [47].

Thermal simulation and testing are indispensable for predicting package temperature fields and validating thermal-management designs [45]. Figure 18 presents a representative chip thermal test; its electrical response is used to infer temperature-dependent loss and thermal resistance.

(1) Finite Element Analysis (FEA)

By using FEA software such as ANSYS Mechanical 2024 R2 and COMSOL Multiphysics 6.2, the thermal distribution inside the package can be simulated, the impact of thermal stress on the package can be predicted, and design solutions can be optimized. Through simulation, designers can identify thermal hotspots in advance and implement effective cooling solutions [48,57].

(2) Thermal imaging technology

Thermal imaging technology is a non-contact method that captures the temperature distribution on the package surface using an infrared camera, providing a visual display of the surface temperature distribution and hotspot locations. In practical applications, thermal imaging technology can be used to detect the thermal uniformity of the package and make targeted optimizations [58].

(3) Thermal resistance model

By establishing an equivalent thermal resistance model of the packaging system, the coupling relationship between temperature, device parameters, and power consumption can be quantitatively described. As shown in Figure 18, the device resistance has an approximately linear relationship with temperature, and the power loss resulting from this relationship increases significantly with packaging temperature. The thermal resistance and loss model fitted based on experimental data can quickly predict the temperature rise and power consumption evolution process inside the package without relying on complex numerical simulations, providing effective guidance for the thermal design and reliability assessment of three-dimensional integrated circuits [48].

### 4.2. Mechanical Stress and Mechanical Reliability

In advanced packaging, multi-material stacking and 3D integration inevitably introduce the issue of coefficient of thermal expansion (CTE) mismatch, leading to thermal stress, warping, and interconnection failures [59,60,61]. In addition, in applications such as automotive electronics and aerospace, the packaging must also withstand vibration and shock loads, further exacerbating mechanical reliability challenges [62,63,64].

#### 4.2.1. Sources of Mechanical Stress

(1) Thermal stress

Due to the differing coefficients of thermal expansion (CTE) of different materials within the package, relative motion between materials occurs when the package is exposed to temperature changes, leading to stress [65]. For example, the CTE mismatch between the chip and the substrate is one of the main causes of thermalmechanical reliability issues in packaging [59,60]. This stress can lead to cracking of the package or failure of the contact between the chip and the substrate.

(2) Mechanical shock and vibration

**Figure 19:**
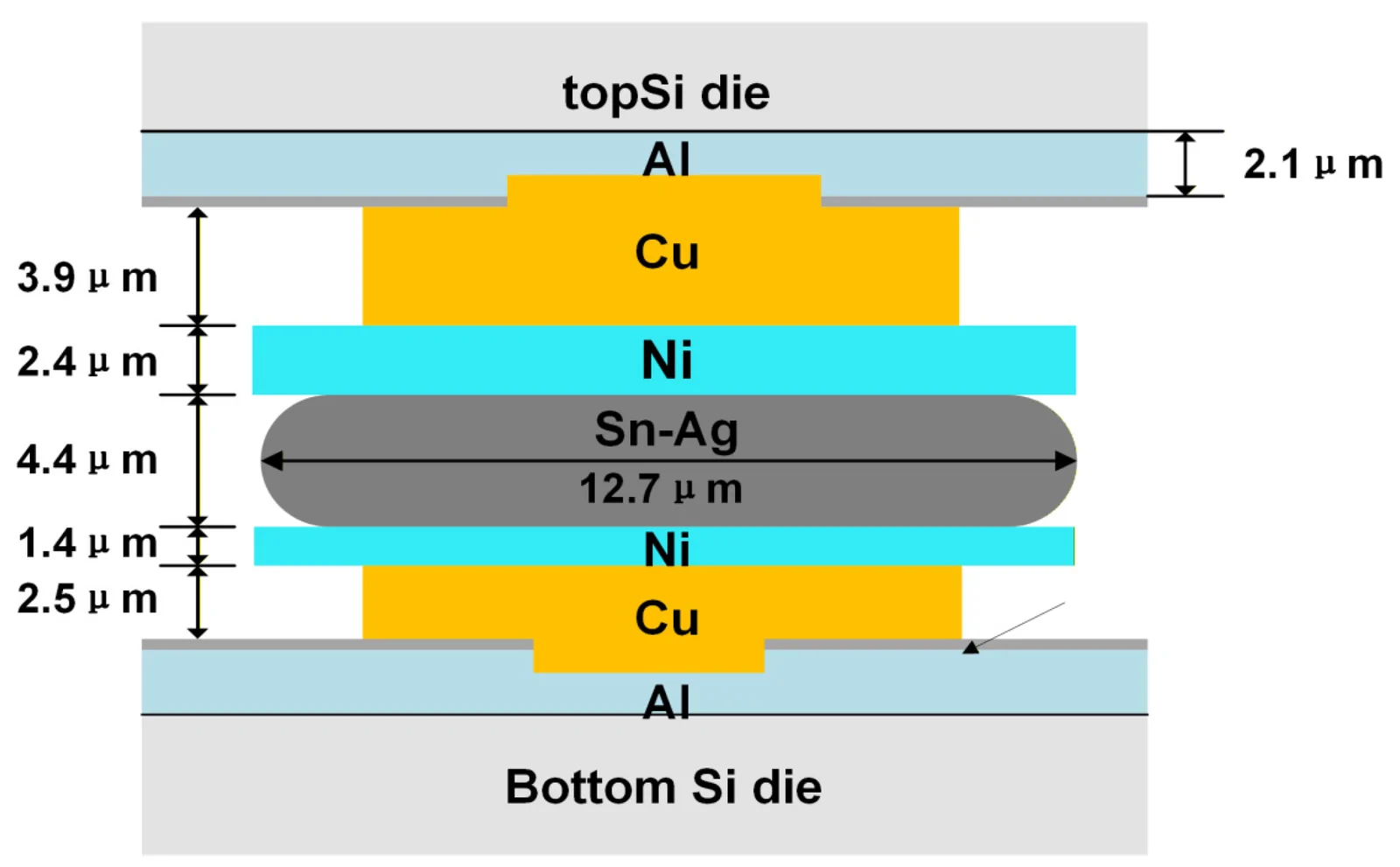
Schematic diagram of micro-bump structure in packaging Adapted from Yao et al. [61].

During the operation of electronic devices, they are often subjected to mechanical shock and vibration, especially in fields such as automotive electronics and aerospace. Under the influence of these external forces, as shown in Figure 19, the solder joints, bumps, and chips within the package may experience damage, affecting the long-term stability of the device [62,66,67].

#### 4.2.2. Reliability Enhancement Strategies

(1) Material and interface optimization

Selecting materials with compatible coefficients of thermal expansion (CTEs) and controlling interface adhesion can reduce warpage and interfacial stress [68]. In flip-chip ball grid array (FCBGA) packages, epoxy-phenolic build-up films and photosensitive polyimide (PSPI) layers can be engineered as dielectric and stress-buffer layers [69]. Low-temperature Ag–Ag bonding based on plasma-induced Ag_2_O nanoparticles has demonstrated void-free, high-strength microconnections at 220 °C [70]. The hardness of nanoparticle-deposited gold-cone microbumps decreases with temperature, reducing the pressure required for elevated-temperature bonding; this relationship must be incorporated into compression and joint-height control [71].

(2) Structural and layout design

**Figure 20:**
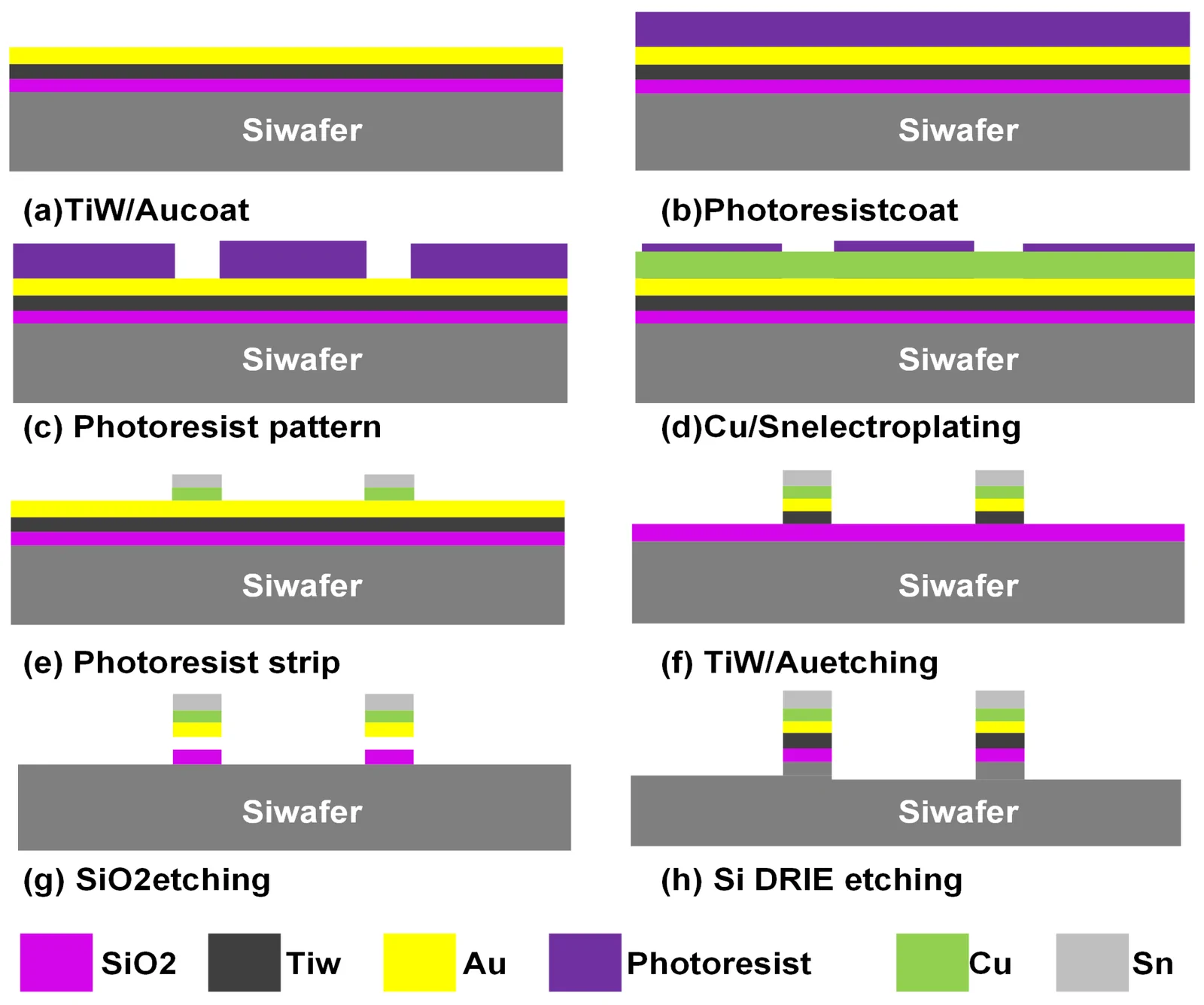
The typical microbump structure and its relevance to mechanical reliability in advanced packaging.

Through the co-optimization of structure and process, the mechanical reliability of advanced packaging can be significantly improved, reducing warping and stress concentration caused by coefficient of thermal expansion (CTE) mismatch. As shown in Figure 20, the formation of microbumps typically involves key steps such as TiW/Au seed layer deposition, photolithography, electroplating Cu/Sn, de-resist, and deep reactive ion etching (DRIE), and their geometric shape and interface quality directly affect the subsequent packaging’s stress distribution and bonding reliability.

In 2.5D and Fan-Out Chip on Substrate (FOCoS) packaging, the chip-last structure of FOCoS, by introducing an underfill layer, can alleviate the CTE mismatch between the chip and the substrate to some extent, thus showing lower overall warpage [72]. At the same time, the planar-to-bump microbump interface design enhances local contact pressure during the bonding process, promoting lateral plastic deformation of the microbumps, thereby improving bonding strength and reliability [73]. In addition, introducing a buffer layer or stress-relief structure between the chip and substrate also helps absorb and redistribute thermal stress [74].

For Fan-Out Wafer-Level Packaging (FOWLP) and 2.5D packaging, the redistribution layer (RDL) can, to some extent, act as a stress-buffering layer, reducing the stress on the extremely low dielectric constant (ELK) interconnect layer. However, in the FOCoS chip-last structure, due to the introduction of high-modulus underfill materials, the stress between the ELK layer and RDL routing may actually be higher than that in the chip-first configuration, with the maximum stress typically concentrated in the gap region between the ASIC and HBM chips. Studies have shown that appropriately reducing the PI/RDL layer thickness is one of the most effective ways to alleviate this type of stress concentration [72].

In wafer-level packaging (WLP), optimizing the PBO-based RDL structure is also crucial. By properly designing the RDL landing positions and protecting the seal ring structure, stress concentration in the hotspot areas can be effectively reduced, thereby significantly improving the reliability of chip-package interconnects (CPI) [75]. Overall, combining microbump manufacturing processes, RDL structure design, and material parameter optimization to co-control stress from both process and structural perspectives is the key approach to enhancing the mechanical reliability of advanced packaging.

(3) Stress modeling and prediction

Finite Element Analysis (FEA): FEA is an important tool for predicting stress distribution and deformation behavior in packaging. By establishing a detailed 3D simulation model, the package’s response under conditions such as thermal cycling and mechanical shock can be simulated, allowing the identification of stress concentration areas and potential failure points. For multi-chip packaging, the EMC volume is the dominant factor influencing warpage, and accurately modeling the actual height and planar dimensions of all passive components is crucial for improving warpage prediction accuracy [76,77]. In addition, by embedding silicon-based piezoresistive stress sensors for in situ monitoring of stress evolution in advanced packaging structures such as Fan-Out Wafer-Level Packaging (FOWLP), stress accumulation phenomena that traditional methods cannot observe can be detected and can be used to calibrate FE models for more precise reliability design [75].

**Figure 21:**
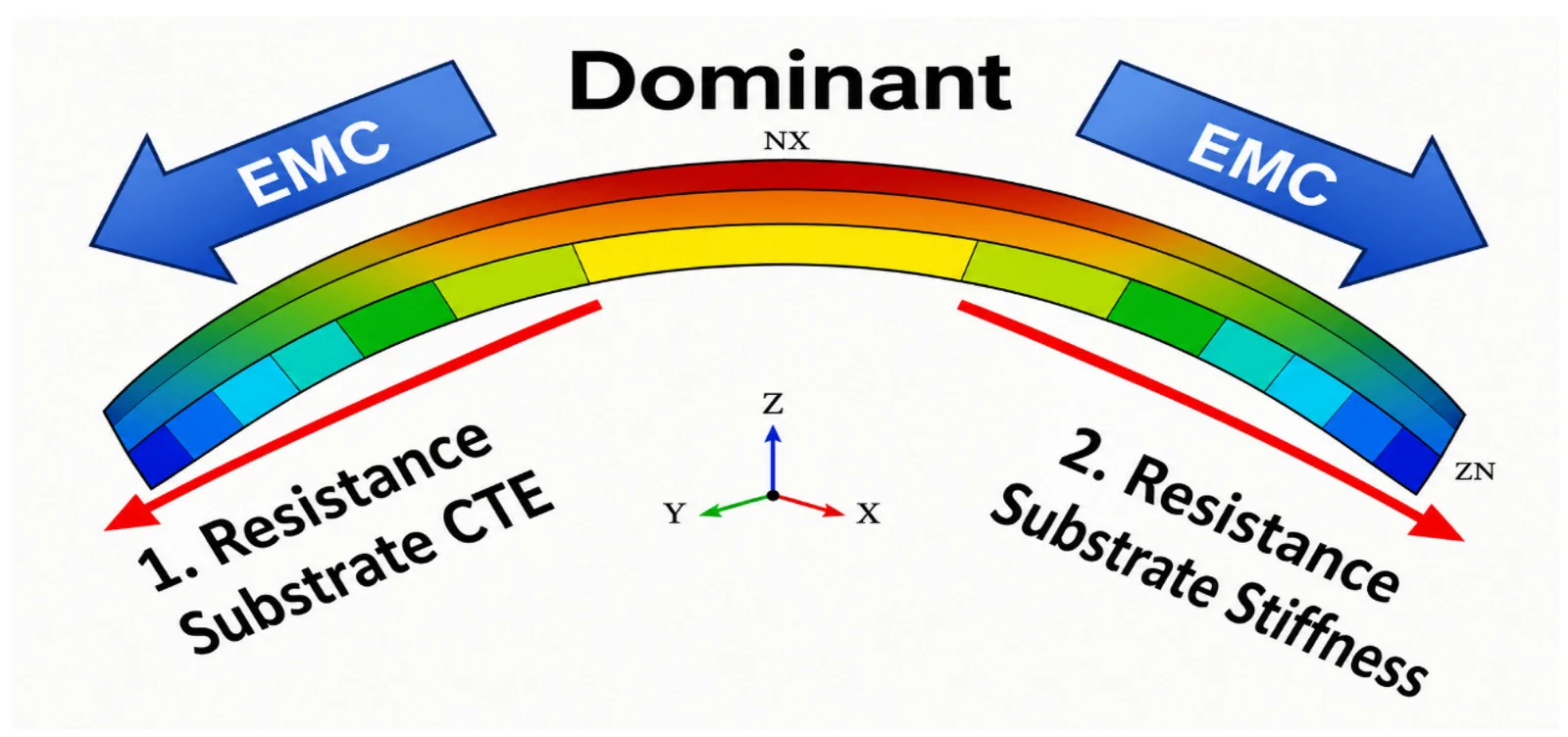
Factors leading to warpage behavior mechanisms: the color gradient indicates the relative dominance of the contributing mechanisms; NX and ZN denote the directional/material-property cases labeled in the source figure. Adapted from Kim et al. [46].

Warpage analysis: Warpage is a common manifestation of thermal stress in packaging. As shown in Figure 21, through thermo-mechanical simulation and experimental measurements, the impact of different packaging structures (such as Fan-Out Panel-Level Packaging) and material parameters on warpage can be analyzed, guiding design optimization to ensure flatness during manufacturing and usage [78,79,80,81,82]. For example, combining FE simulation with deep learning models (such as ResNet-152 or GCVIT-Tiny) can efficiently and accurately predict FOWLP wafer warpage. In addition, the data-driven stress/warpage analysis method, combining Stoney’s equation with artificial neural networks (ANNs), can handle structures that involve complex factors such as multilayer films, thick films, and viscoelastic films and has the potential to be extended to 3D warpage topology reconstruction with discrete film segments [83].

### 4.3. Electrical Performance and Electrical Reliability

As packaging density and operating frequency continue to increase, electrical reliability issues have become more prominent, mainly manifested in signal integrity (SI), power integrity (PI), and electromagnetic interference (EMI) [84,85].

#### 4.3.1. Signal Integrity (SI)

Signal integrity (SI) focuses on the quality of signal transmission in packaging and interconnection structures, mainly manifested in amplitude attenuation, timing distortion, and crosstalk. As the integration density of advanced packaging continues to increase, although signal paths are significantly shortened, high-density interconnections and high-frequency operating conditions also make noise coupling and reflection issues more prominent, thus posing challenges to system performance.

To ensure stable transmission of high-speed signals, impedance matching is a key factor in packaging design. By appropriately designing the routing geometry, interlayer structure, and reference planes, the transmission line impedance can be kept continuous, effectively suppressing signal reflection, reducing eye diagram degradation, and improving the signal quality of high-speed interfaces [86].

In addition, the introduction of low dielectric constant materials is crucial for improving high-frequency signal transmission. Using packaging substrates and dielectric layers with low dielectric constants and low loss factors can significantly reduce dielectric loss and propagation delay, thereby enhancing the transmission performance of high-speed and millimeter-wave signals to meet the signal integrity requirements of advanced packaging in high-frequency applications [87].

#### 4.3.2. Power Integrity (PI)

Power integrity (PI) is a core factor in ensuring the stable operation of high-speed, high-power systems, and its essence lies in suppressing voltage fluctuations and noise in the power delivery network (PDN). The PDN exhibits distinct capacitive and inductive characteristics across different frequency ranges, and resonance peaks may occur in the mid-to-high frequency range, leading to voltage fluctuations and power concentration, which can further cause localized temperature rise.

**Figure 22:**
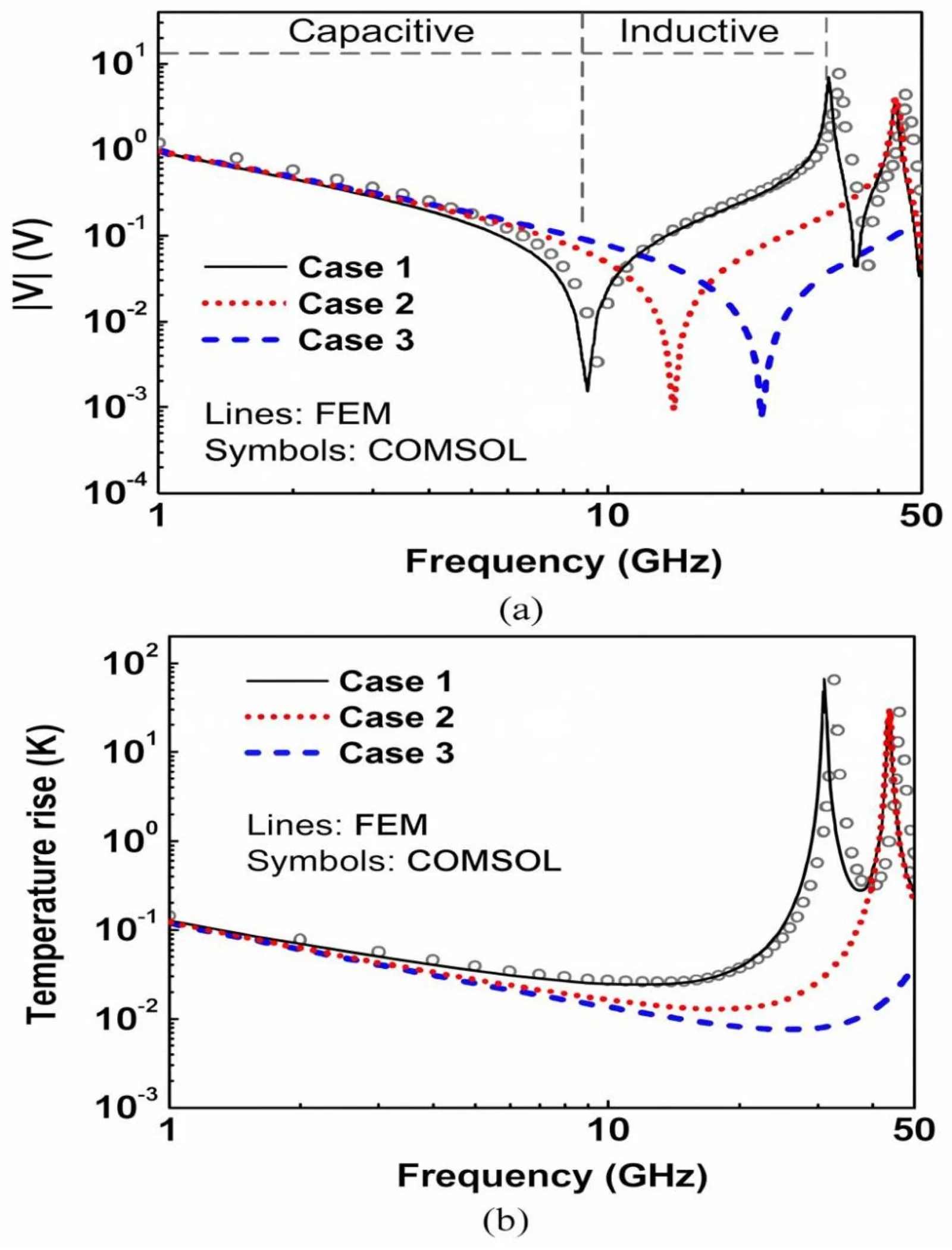
Influence of decoupling and layout optimization on power integrity performance: (**a**) impedance magnitude versus frequency in capacitive and inductive regions; (**b**) temperature rise versus frequency. Solid lines denote FEM results, symbols denote COMSOL results, and the dashed/dotted curves distinguish the three cases. Adapted from Li et al. [87].

To enhance PI performance, proper placement of decoupling capacitors is crucial. By placing high-frequency decoupling capacitors near the chip power bumps, high-frequency impedance can be effectively reduced, and synchronous switching noise can be suppressed, thereby improving transient voltage response. Different design cases in Figure 22 show that optimizing decoupling strategies can significantly reduce resonance peaks, minimize voltage fluctuations, and their corresponding temperature rise.

In addition, optimizing the power path and layout is a key means of reducing parasitic parameters in the PDN. By shortening the power supply loop and reducing the parasitic resistance and inductance between power and ground, voltage drops can be effectively reduced, and system stability can be enhanced [88]. Under high current density conditions, a well-designed PDN also helps avoid local current congestion, thus reducing the risk of electromigration (EM) failures and improving the long-term reliability of the system [89].

#### 4.3.3. Vertical Power Delivery

Vertical power delivery moves part of the power-delivery network (PDN) from long lateral frontside routes to backside, interposer, bridge, or vertically integrated paths. Backside PDNs separate signal and power routing and can reduce frontside congestion and resistive voltage drop. Published benchmarking shows that the benefit depends strongly on the contact scheme and via resistance; direct-backside-contact approaches provide stronger cell-level scaling, whereas some deep-via schemes remain limited by vertical resistance [90,91]. At package level, pass-through TSVs, power-capable bridges, embedded decoupling capacitors, and vertically integrated regulators shorten the path between package power entry and high-current compute dies.

The electrical benefit must be assessed together with thermal and mechanical consequences. Backside metallization and power vias alter the heat-flow path, additional interfaces introduce stress and alignment constraints, and vertically integrated regulators can create local heat sources. Qualification should therefore report DC IR drop, frequency-dependent target impedance, transient voltage droop, current density, via/contact resistance, temperature rise, and reliability under combined current and thermal cycling. Vertical power delivery is most valuable when these package-level measurements demonstrate a net system benefit rather than only a reduction in on-die routing area.

#### 4.3.4. Electromagnetic Interference (EMI)

Electromagnetic interference (EMI) is a common issue in modern electronic systems. As packaging density increases, electromagnetic noise also rises, potentially interfering with other sensitive components [92]. Effective electromagnetic shielding and layout optimization are key to reducing the impact of EMI [93].

Introducing electromagnetic shielding layers into packaging design is an effective method to isolate noise sources from sensitive components and reduce electromagnetic interference propagation. For example, magnetoelectric (ME) dipole antennas can effectively suppress electromagnetic radiation by integrating shielding structures within the package, especially in space-constrained portable devices. In addition, using a non-uniform through-glass vias (TGV) design can enhance electromagnetic shielding effects by optimizing grounding references while ensuring signal integrity [94].

**Figure 23:**
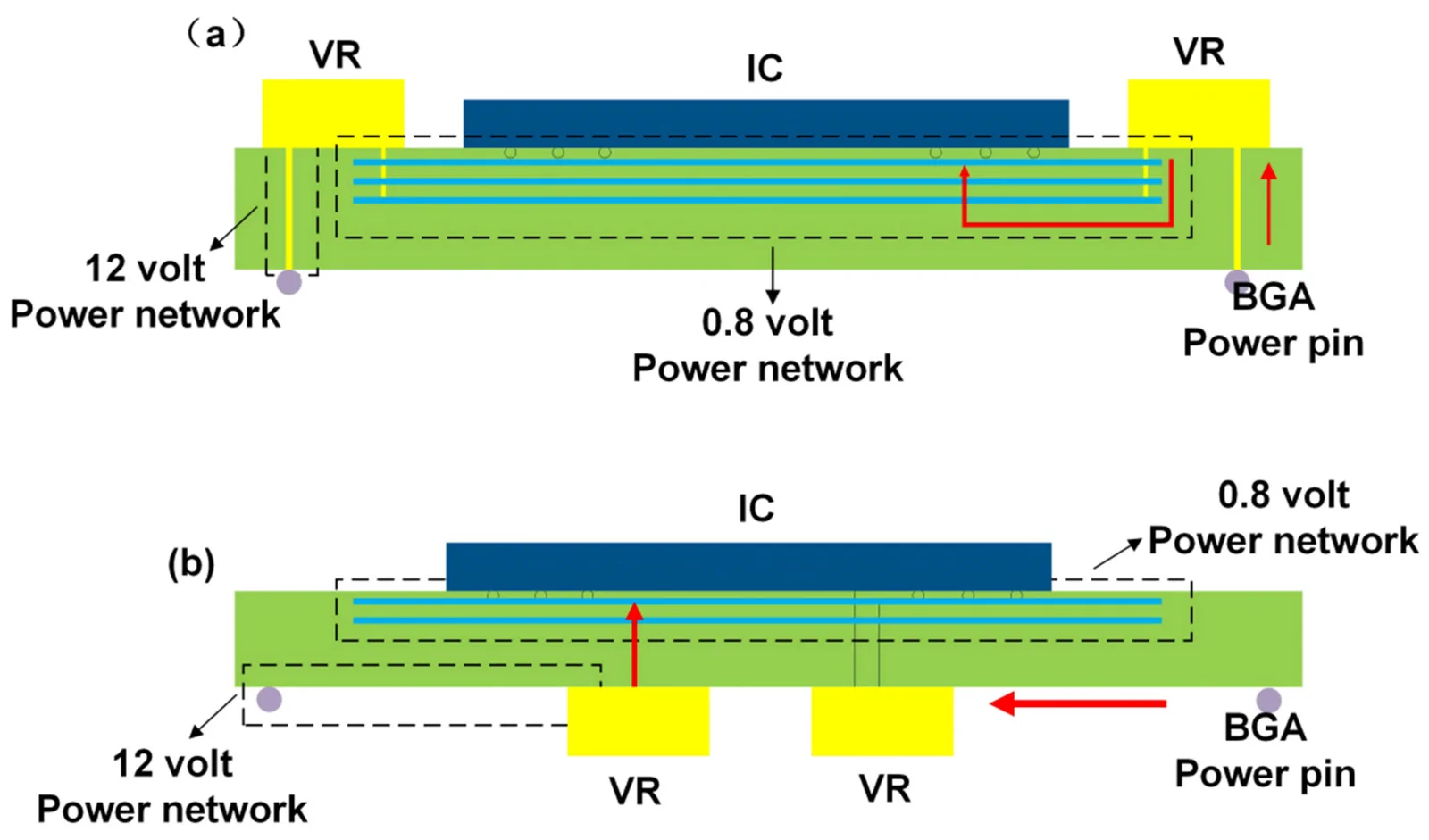
VR topology structure in SiP (**a**) Top layer structure (**b**) Bottom layer structure Adapted from Wang et al. [7].

By partitioning different functional modules, such as separating high-frequency digital signals from low-frequency analog signals, mutual interference between them can be reduced. In addition, optimizing the length and spacing of interconnections can reduce crosstalk, which is a major challenge in high-density interconnect technologies [95]. As shown in Figure 23, in RF systems, using constant V-shaped wire bonding techniques can provide stable grounding for low-noise amplifiers (LNAs), effectively reducing inductance and signal crosstalk [96]. Additional package-level EMI studies address shielding, grounding, interconnect coupling, and high-frequency co-design [97,98,99,100,101,102].

## 5. Conclusions and Outlook

### 5.1. Conclusions

**Figure 24:**
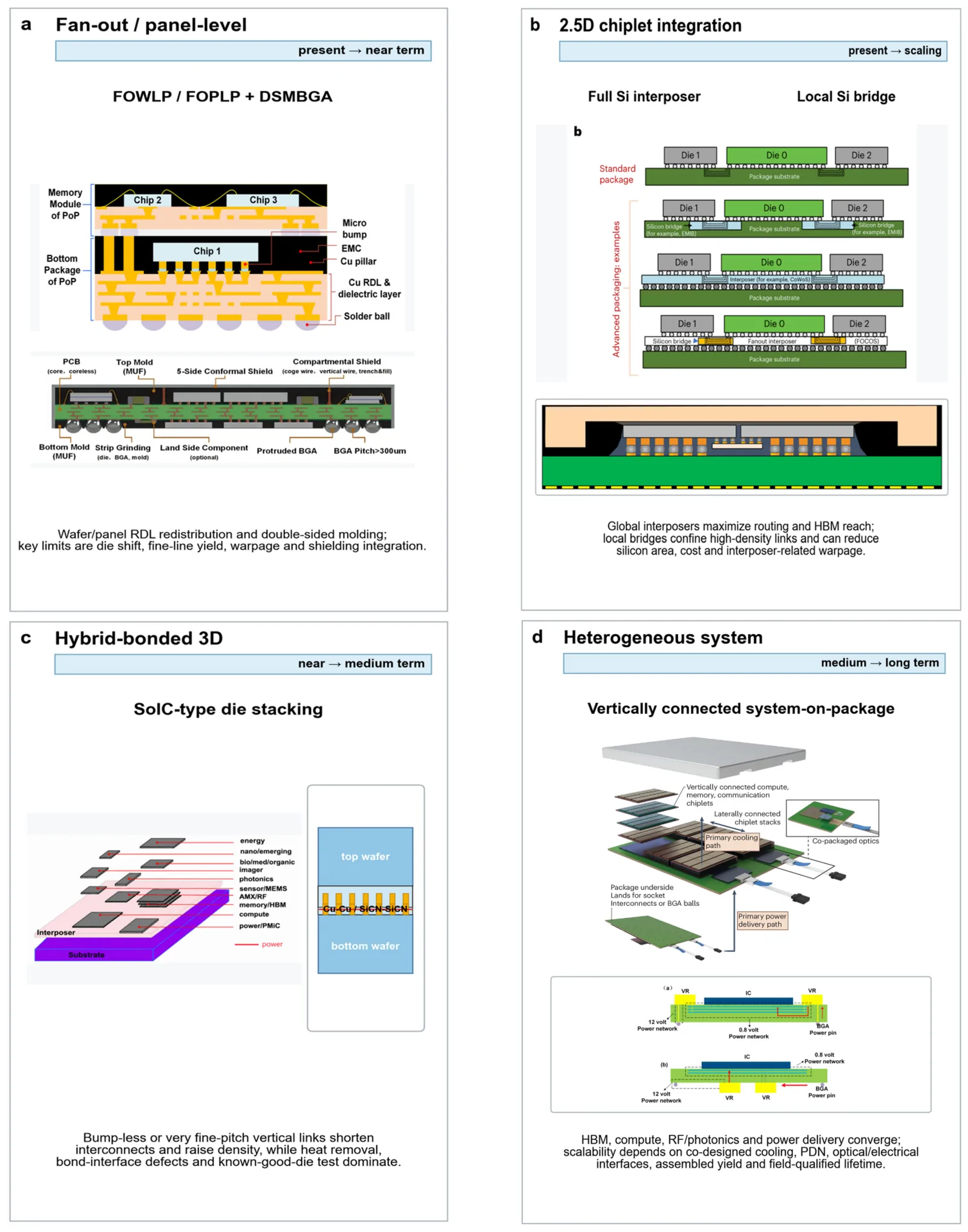
(**a**). Fan-out and panel-level packaging architectures integrating FOWLP, FOPLP, and DSMBGA; (**b**). 2.5D chiplet integration using full silicon interposers and local silicon bridges; (**c**). Hybrid-bonded 3D integration based on SoIC-type die stacking; (**d**). Heterogeneous system-on-package integrating chiplets, co-packaged optics, cooling, and power delivery. Panel (**a**) is based on refs. [7,12,30]; panel (**b**) on refs. [7,18,19,20,21,22,23,24]; panel (**c**) on ref. [31]; and panel (**d**) on refs. [7,27,90,91,103].

As shown in Figure 24, advanced packaging should be selected by the location of the system bottleneck. Mature 2D SiP remains appropriate when cost, yield, and planar integration dominate. Full silicon interposers are justified when global routing density and logic-to-HBM bandwidth outweigh interposer cost. Localized bridges provide dense edge connectivity without a package-wide interposer, whereas fan-out RDL platforms prioritize thin form factor and routing flexibility. Three-dimensional stacking provides the shortest interconnects but imposes the strongest constraints on pre-bond testing, heat removal, and coupled reliability.

The comparison also shows that no single density metric predicts system value. Pitch, bandwidth density, energy per bit, current delivery, junction temperature, warpage, assembly yield, and cost must be reported together and under stated conditions. The dominant barriers to larger systems are increasingly package-scale power delivery, heat spreading, multi-die yield, interface qualification, and design-tool convergence rather than the availability of an isolated fine-pitch interconnect process.

### 5.2. Development Trends and Outlook

The resulting roadmap is summarized in Table 5. Near-term progress is expected from qualified bridge/RDL scaling, improved HBM integration, backside and vertical power delivery, glass-core evaluation, and multiphysics sign-off. Medium-term systems will require broader hybrid-bonding deployment, co-designed cooling and PDNs, interoperable chiplet interfaces, and predictive reliability models. Long-term scaling depends on repairable or redundancy-aware multi-die assemblies, integrated photonic/RF links, closed-loop thermal and power management, and manufacturing flows that control material and energy intensity.

For 5G/6G and radar systems, the priority is not maximum digital interconnect density but low-loss materials, antenna-in-package integration, calibration stability, isolation, and heat removal from compound-semiconductor devices. For AI and HPC, the priority is sustained logic-memory bandwidth at acceptable energy per bit while delivering hundreds of amperes and removing package-scale heat. Automotive adoption requires these benefits to survive mission-profile temperature, humidity, shock, vibration, and extended service life.

Glass-core substrates and glass interposers offer dimensional stability, tunable electrical properties, and potential large-format processing, but through-glass-via formation, metallization adhesion, panel handling, and ecosystem maturity remain qualification barriers. Hybrid bonding can reduce interconnect pitch and capacitance, but surface planarity, contamination control, alignment, known-good-die testing, and post-bond repairability determine whether laboratory pitch scaling becomes manufacturable system scaling.

AI-assisted electronic design automation (EDA) is likely to be most useful when it accelerates validated multiphysics analysis rather than replacing physics-based qualification. Surrogate models can screen large design spaces for temperature, warpage, electromigration, and signal/power integrity, but uncertainty bounds, out-of-distribution detection, and confirmation against finite-element models and physical test vehicles are required before reliability decisions are automated.

The architecture-level implications of Table 5 are illustrated in Figure 24. Near-term scaling is expected to be led by fan-out/panel-level and 2.5D platforms, whereas hybrid-bonded 3D integration and heterogeneous system-on-package architectures become increasingly important over the medium and long term. Across this progression, interconnect density, power delivery, thermal removal, assembly yield, and qualification must be co-optimized rather than treated independently.

Overall, the field is moving from package assembly toward system technology co-optimization. Progress should be judged by reproducible improvements in performance, power, area, cost, yield, and qualified lifetime. Reporting these quantities with consistent boundary conditions will make comparisons more useful to both researchers and engineers and will help distinguish scalable architectures from isolated demonstrations.

## Figures and Tables

**Table 1:** Baseline comparison of 2D, 2.5D, and 3D advanced packaging architectures.

Technology	Structure/Representative Scale	Applications	Quantitative Characteristics
2D SiP	Planar organic-substrate integration; UCIe-S bump pitch 100–130 µm and channel reach <25 mm	Mobile devices, IoT, RF modules	UCIe-S target: 28–224 GB/s/mm shoreline bandwidth and ~0.5 pJ/bit; mature process and yield, but lowest interconnect density
2.5D SiP	Si interposer, localized bridge, or advanced fan-out; UCIe-A bump pitch 25–55 µm and reach <2 mm	HPC, AI accelerators, GPUs	UCIe-A target: 165–1317 GB/s/mm and ~0.25 pJ/bit; CoWoS-S interposer up to ~2700 mm^2^ (3.3× reticle) [21]
3D SiP	Vertical microbump or Cu-Cu hybrid-bonded stacking; SoIC starts at sub-10 µm bond pitch	AI, high-speed computing, RF systems	UCIe-3D illustrative 9 µm point: ~4 TB/s/mm^2^ and <0.05 pJ/bit; shortest links but strongest thermal and pre-bond-test constraints

**Table 2:** Common comparison framework for representative advanced packaging architectures.

Architecture	Interconnect/Reported Pitch	Representative Adoption	Manufacturing and Cost Constraint	Dominant Reliability Limits	Quantified Scaling Reference
2D organic-substrate SiP	UCIe-S: 100–130 µm bump pitch; <25 mm reach	Mobile, RF, IoT	Substrate layers and board area	Solder fatigue, delamination, EMI	28–224 GB/s/mm and ~0.5 pJ/bit UCIe-S target
Full Si interposer + TSV	45 µm microbumps and 1200 mm^2^ CoWoS-2 test vehicle [16]	CoWoS-S-class HPC/AI + HBM	Interposer area, TSV process, assembly yield	Microbump fatigue, interposer stress, hotspots	UCIe-A: 165–1317 GB/s/mm and ~0.25 pJ/bit
Localized Si bridge	30 μm reported for IBM DBHi test vehicle [22]; Intel reports 45–55 μm generations [24]	EMIB; CoWoS-L/LSI; DBHi research	Bridge placement, cavities, local underfill, ecosystem	Bridge-edge stress, local joint fatigue, warpage	Qualify ≤ 30 µm-class pitch with multi-bridge yield and vertical power [22]
RDL fan-out	Reported lithography: 0.8–1.5 µm L/S; 1.5–2.0 µm microvias at ≤5 µm pitch	InFO and fan-out SiP	Placement/overlay, mold shrinkage, wafer/panel yield	RDL cracking, warpage, electromigration	1 µm-class RDL and 5–10 µm I/O pitch test vehicles with controlled overlay/warpage
3D microbump stacking	Advanced-package range 25–55 µm; product and PHY dependent	Logic-memory and logic-on-logic stacks	Stack yield, test access, thermal path	Hotspots, TSV stress, microjoint fatigue	Move below 25 µm only with known-good-die and thermal co-design
3D hybrid bonding	Sub-10 μm class publicly demonstrated in advanced platforms; interface-specific [25]	SoIC/Foveros Direct-class integration	Surface planarity, contamination, alignment, repairability	Bond voids, Cu diffusion/stress, thermal coupling	Illustrative 3 µm UCIe-3D point: ~35 TB/s/mm^2^ and <0.02 pJ/bit

**Table 3:** Application requirements, preferred architectures, and residual bottlenecks.

Application	Primary Requirement	Preferred Architecture	System-Level Benefit	Residual Bottleneck/Qualification Need
Power modules	High current and low inductance; 900 V/196 A module characterized to 200 °C	Embedded/double-sided cooled power module; 2.5D routing where needed	Double-sided cooling example: Rth = 0.51 °C/W, 28.5% below single-sided cooling	Interface aging, insulation, power cycling, vibration
HPC and AI	Up to 700 W accelerator TDP; HBM bandwidth 3.35–4.8 TB/s in H100/H200 examples	Full interposer or RDL/bridge platform; 3D stacking for selected tiers	H200 example: 141 GB HBM3e at 4.8 TB/s and 900 GB/s NVLink	Package-scale heat spreading, warpage, yield, cost
5G/6G and radar	Millimeter-wave front ends; automotive radar at 76–81 GHz with up to 4 GHz bandwidth [26]	Fan-out/AiP/RF SiP; localized heterogeneous integration	At B = 4 GHz, theoretical range resolution c/(2B) is ~3.75 cm; short RF paths reduce parasitics	Material loss, calibration, EMI, high-power GaN cooling
MEMS/photonics	12 × 12 switch example: 25 µm RDL L/S, 50 µm solder spheres, 127 µm fiber pitch [27]	Flip-chip, interposer, wafer-level hermetic or optical package	10 Gb/s, BER 10^−11^, 4.25 dB/facet optical loss, and 0.4 µs switching [27]	Alignment drift, contamination, stress, test access
Automotive	AEC-Q100 ambient ranges: Grade 0, −40 to 150 °C; Grade 1, −40 to 125 °C [28]	Qualified SiP/power module with conservative interconnect rules	Reduced module size and harness complexity; integrated 77–81 GHz radar and power electronics	Mission-profile qualification and repairability

**Table 4:** Architecture-specific reliability mechanisms, evaluation methods, and mitigation strategies.

Architecture	Trigger and Vulnerable Location	Dominant Mechanism	Representative Quantitative Evaluation	Mitigation
Full Si interposer	Power cycling; microbumps and TSV keep-out zones	CTE-driven fatigue, TSV stress, hotspot interaction	Daisy-chain ΔR/R0, warpage (µm), thermography; example TCT −65 to 150 °C/500 cycles and uHAST 130 °C/85% RH/96 h	Underfill/TIM optimization, floorplanning, compliant interfaces
Localized bridge	Local stiffness transition at bridge edges	Joint fatigue, delamination, stress concentration	Four-wire resistance/continuity, cross-section, and DIC/FEA at 30 µm DBHi or 45–55 µm EMIB pitch [22]	Bridge-edge geometry, underfill control, placement/coplanarity control
RDL fan-out	Mold cure and thermal excursions; RDL/via interfaces	Warpage, RDL cracking, electromigration	Warpage (µm), crack length, via resistance, and current stress; TCT/uHAST conditions reported with cycle/time count	Mold/RDL stack optimization, balanced layout, current-density limits
3D stack	High power density and vertical temperature gradient	Thermal coupling, microjoint fatigue, TSV stress	Hotspot ΔT, Rth and sensor error; near-junction microfluidic example reaches 0.286 °C·cm^2^/W [32]	Thermal-aware placement, thinning/TIM control, liquid or near-junction cooling
RF/MEMS SiP	Humidity, vibration, and high-frequency coupling	Corrosion, alignment drift, EMI/crosstalk	S11/S21 and shielding (dB); photonic example: BER 10^−11^, 4.25 dB/facet, 0.4 µs [27]; HAST/vibration	Hermetic barriers, stress isolation, compartmental shielding

**Table 5:** Time-phased scaling roadmap and validation criteria.

Horizon	Technology Direction	Measurable Target	Key Dependency	Required Validation
Near term (1–3 years)	RDL/bridge scaling; HBM integration; backside/vertical PDN; glass test vehicles	Demonstrate and qualify 1–2 µm-class RDL L/S, 5–10 µm I/O pitch test vehicles, 45 µm-class EMIB and sub-10 µm SoIC where flow permits [103,104]	Placement/overlay, power-aware packaging, material supply	Electrical-thermal co-test, warpage, thermal cycling, HAST, yield reporting
Medium term (3–7 years)	Broader hybrid bonding; co-designed cooling; interoperable chiplets; AI-assisted sign-off	UCIe-3D 9 µm illustrative point: ~4 TB/s/mm^2^ and <0.05 pJ/bit; report package hotspot temperature and Rth [103]	Known-good-die test, interface standards, model calibration	Large multi-die test vehicles, mission profiles, uncertainty-qualified surrogate models
Long term (>7 years)	Repairable/redundant multi-die systems; photonic/RF co-packaging; closed-loop management	Illustrative UCIe-3D 3–1 µm scaling: ~35–300 TB/s/mm^2^ and <0.02–0.01 pJ/bit; not a manufacturing commitment [103]	Repair/redundancy architecture, sustainable materials/processes	Field-representative lifetime data, lifecycle energy/material accounting

## Data Availability

No new data were created or analyzed in this study. Data sharing is not applicable to this article.
